# Supplementary material for: Michaelis-Arbuzov-Type Reaction of 1-Imidoalkyltriarylphosphonium Salts with Selected Phosphorus Nucleophiles
Source: Molecules. 2019 Sep 19;24(18):3405. doi: 10.3390/molecules24183405 (PMC6767244; doi:10.3390/molecules24183405)
Supplement: Supplementary file 1 [file molecules-24-03405-s001.pdf]

molecules

Article

Michaelis-Arbuzov-type reaction of 1-imidoalkyltriarylphosphonium salts with selected phosphorus nucleophiles

Jakub Adamek[1,2,*], Anna Węgrzyk-Schlieter[1,2], Klaudia Steć[1,2], Krzysztof Walczak[1] and Karol Erfurt[3]

[1] Department of Organic Chemistry, Bioorganic Chemistry and Biotechnology, Silesian University of Technology, B. Krzywoustego 4, 44-100 Gliwice, Poland; jakub.adamek@polsl.pl, anna.wegrzyk-schlieter@polsl.pl, stec-k@o2.pl, krzysztof.walczak@polsl.pl
[2] Biotechnology Center of Silesian University of Technology, B. Krzywoustego 8, 44-100 Gliwice, Poland
[3] Department of Chemical Organic Technology and Petrochemistry, Silesian University of Technology, B. Krzywoustego 4, 44-100 Gliwice, Poland; karol.erfurt@polsl.pl
* Correspondence: jakub.adamek@polsl.pl; Tel.: +48 032-237-1080; fax: +48 032-237-2094

Supporting information

Table of contents

1. ^{1}H-NMR, ^{13}C-NMR, ^{31}P-NMR, MS and IR spectra of compounds **1** ……..………..........…....S2-S52

2. Comparison of ^{1}H-NMR spectra of 1-imidoalkylphosphonium salt **2a** and 1- imidoalkylphosphonate **1a**……………………………………………...………....………...……S53

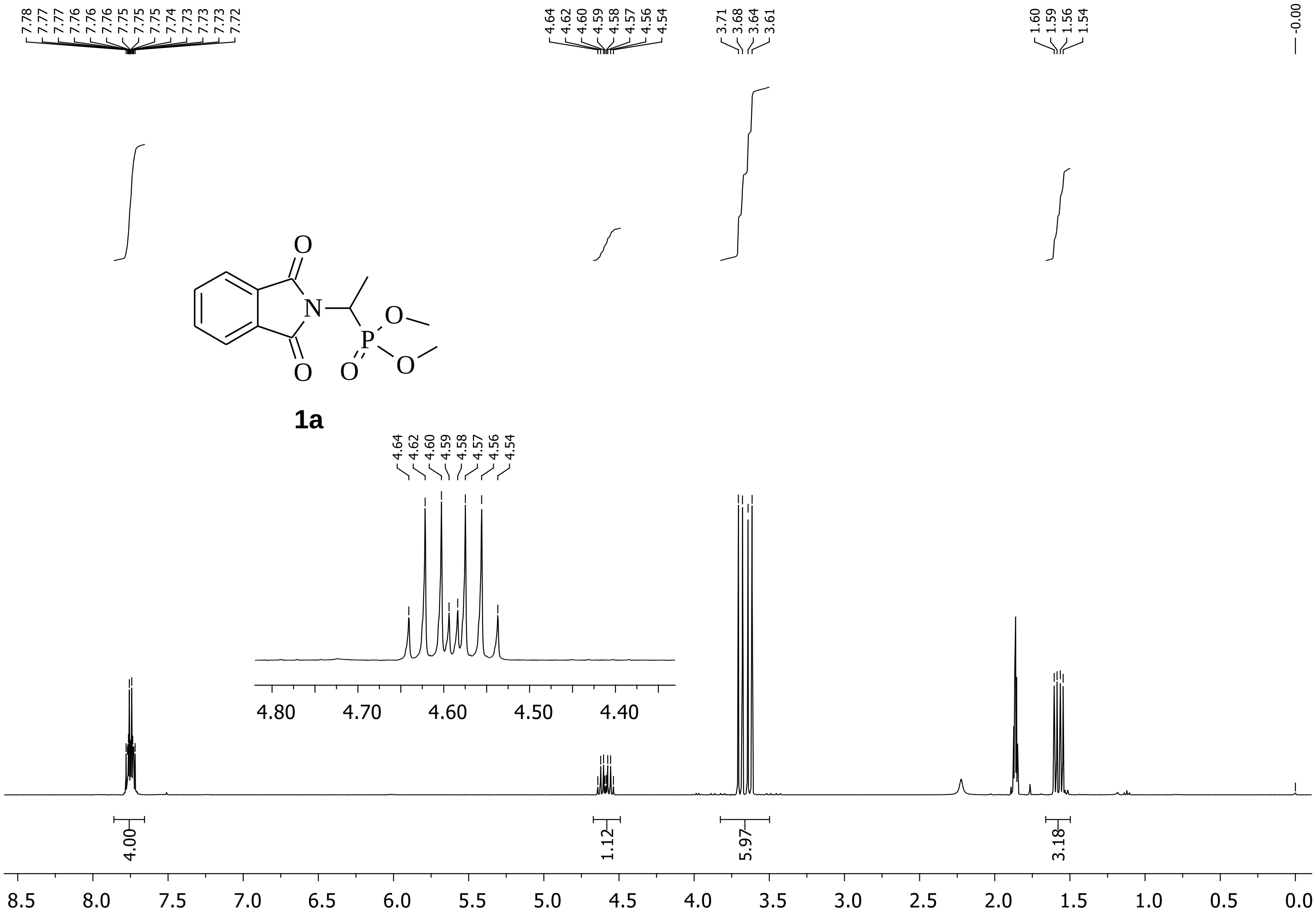

^{1}H-NMR spectrum of dimethyl 1-(*N*-phthalimido)ethylphosphonate (**1a**); 400 MHz/CD_3CN/TMS; δ (ppm).

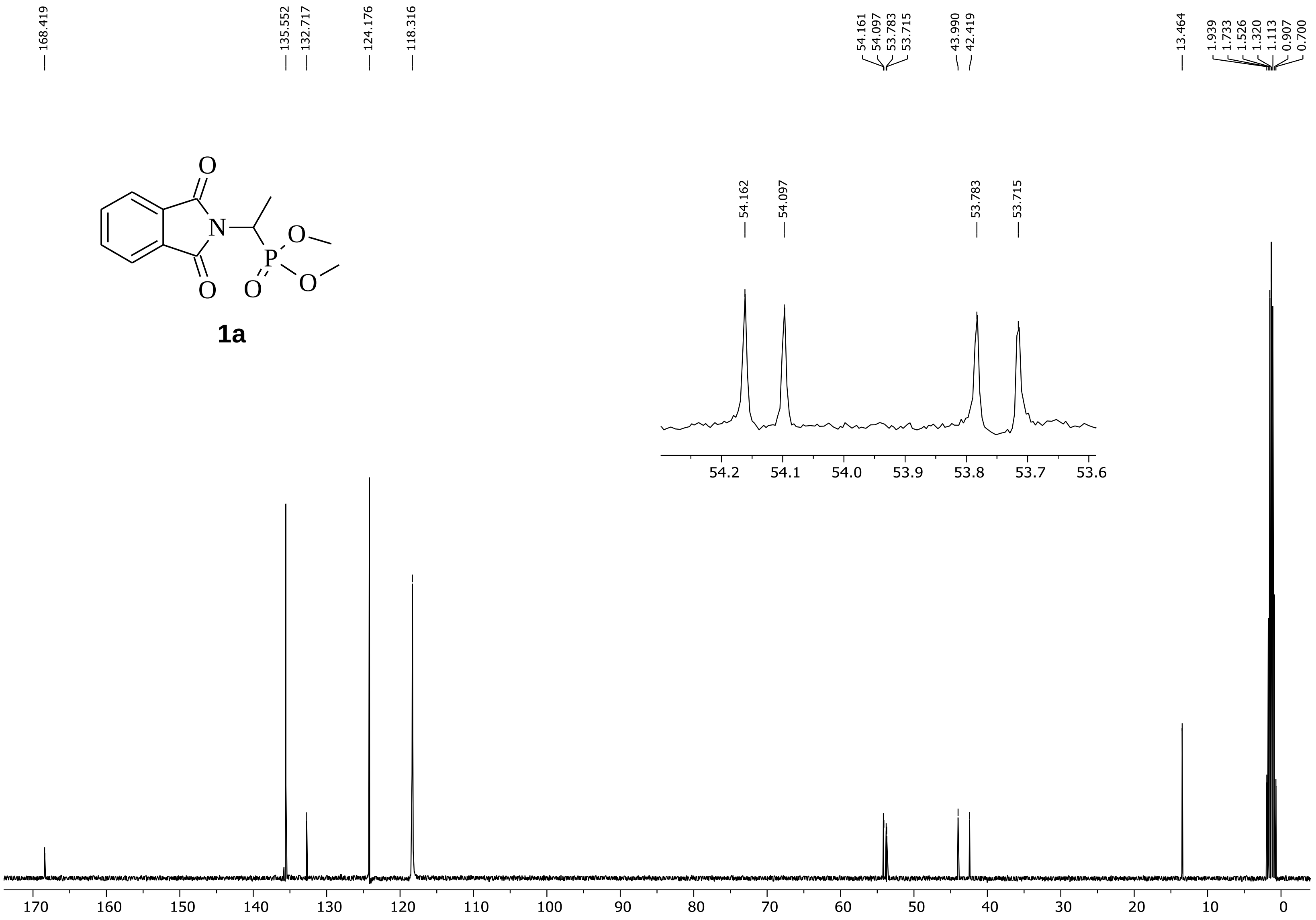

1a

^{13}C-NMR spectrum of dimethyl 1-(*N*-phthalimido)ethylphosphonate (**1a**); 100 MHz/CD_3CN; δ (ppm).

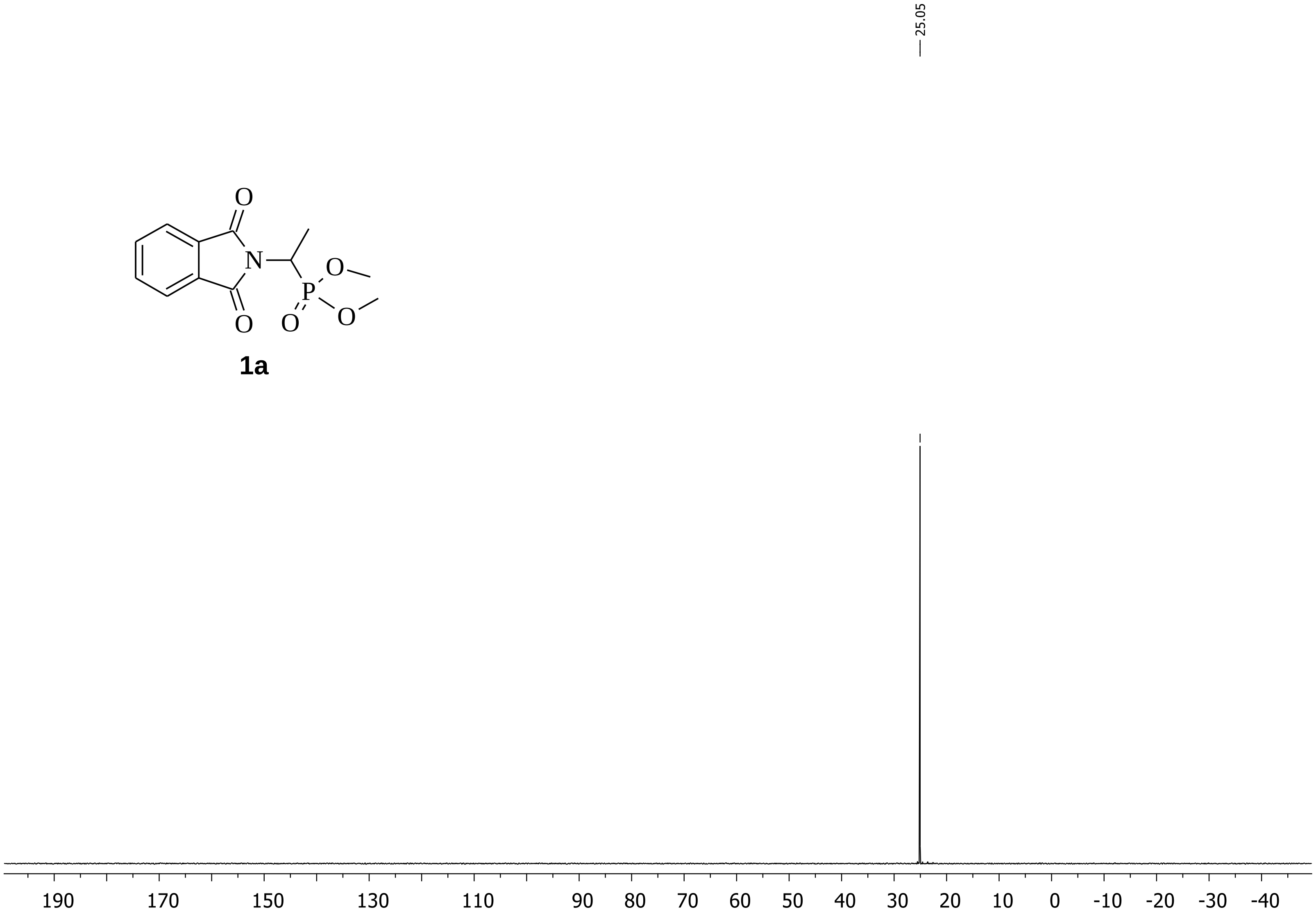

^{31}P-NMR spectrum of dimethyl 1-(*N*-phthalimido)ethylphosphonate (**1a**); 161.9 MHz/CD_3CN; δ (ppm).

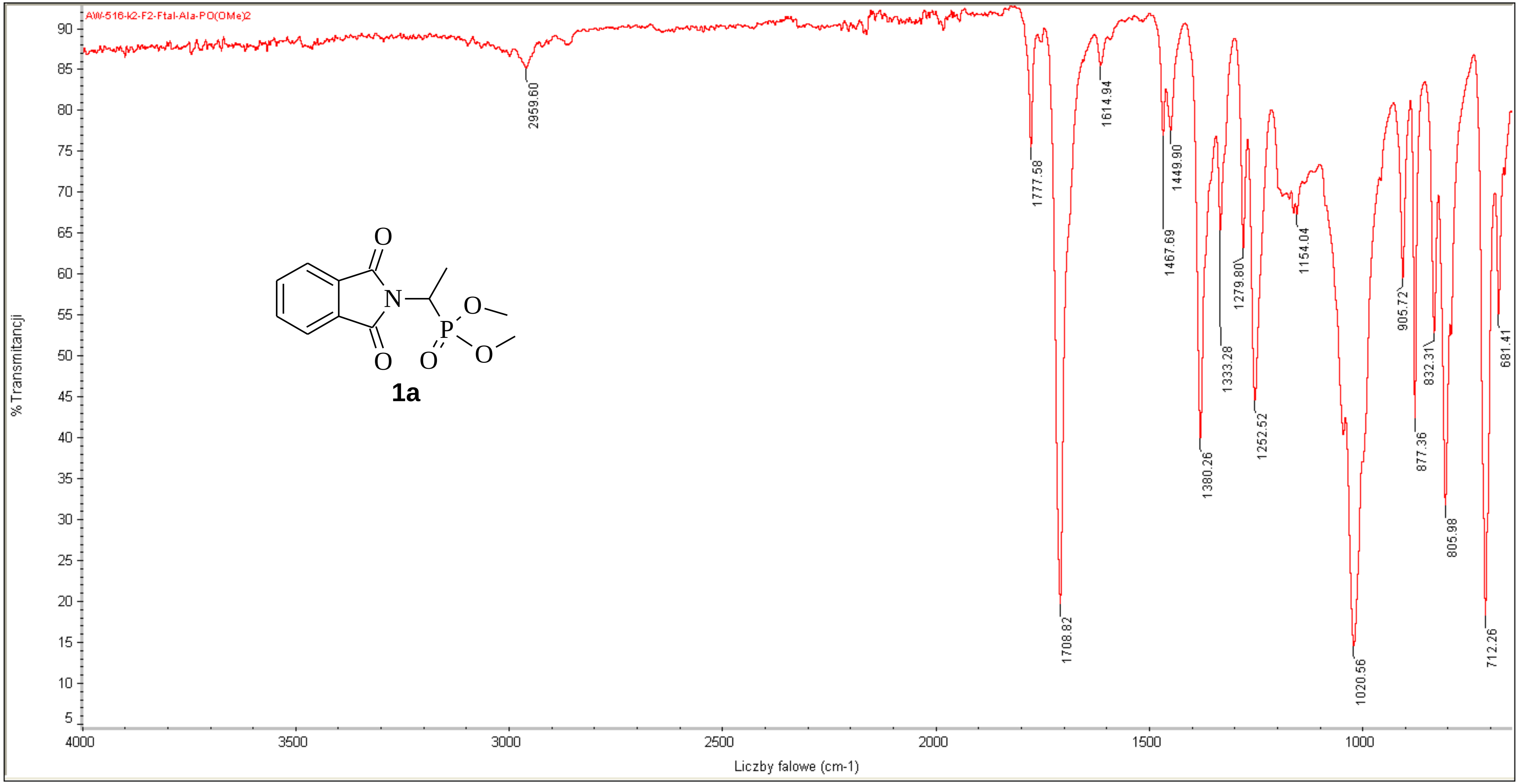

IR spectrum of dimethyl 1-(*N*-phthalimido)ethylphosphonate (**1a**); ATR, cm^{-1}.

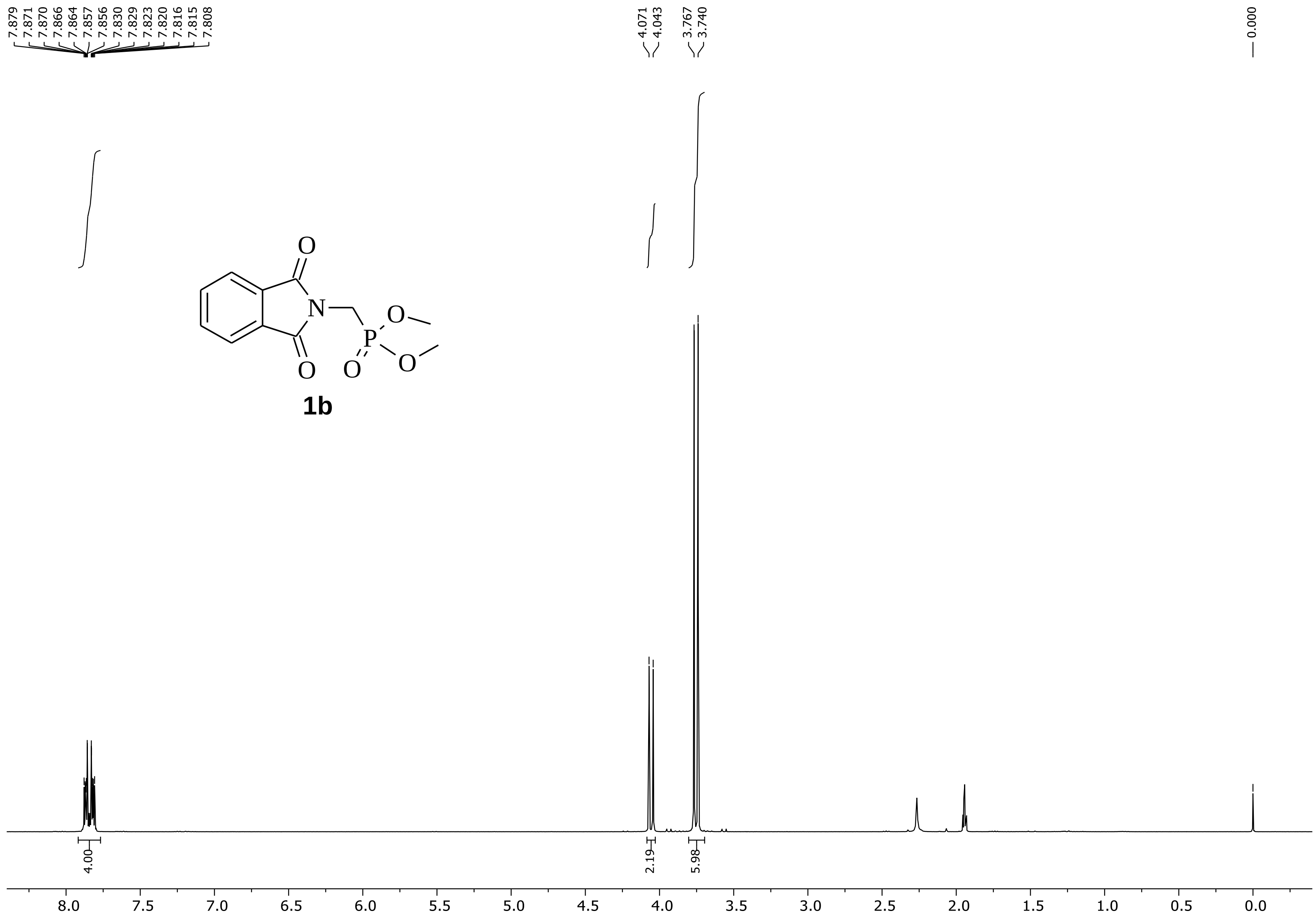

^{1}H-NMR spectrum of dimethyl *N*-phthalimidomethylphosphonate (**1b**); 400 MHz/CD_3CN/TMS; δ (ppm).

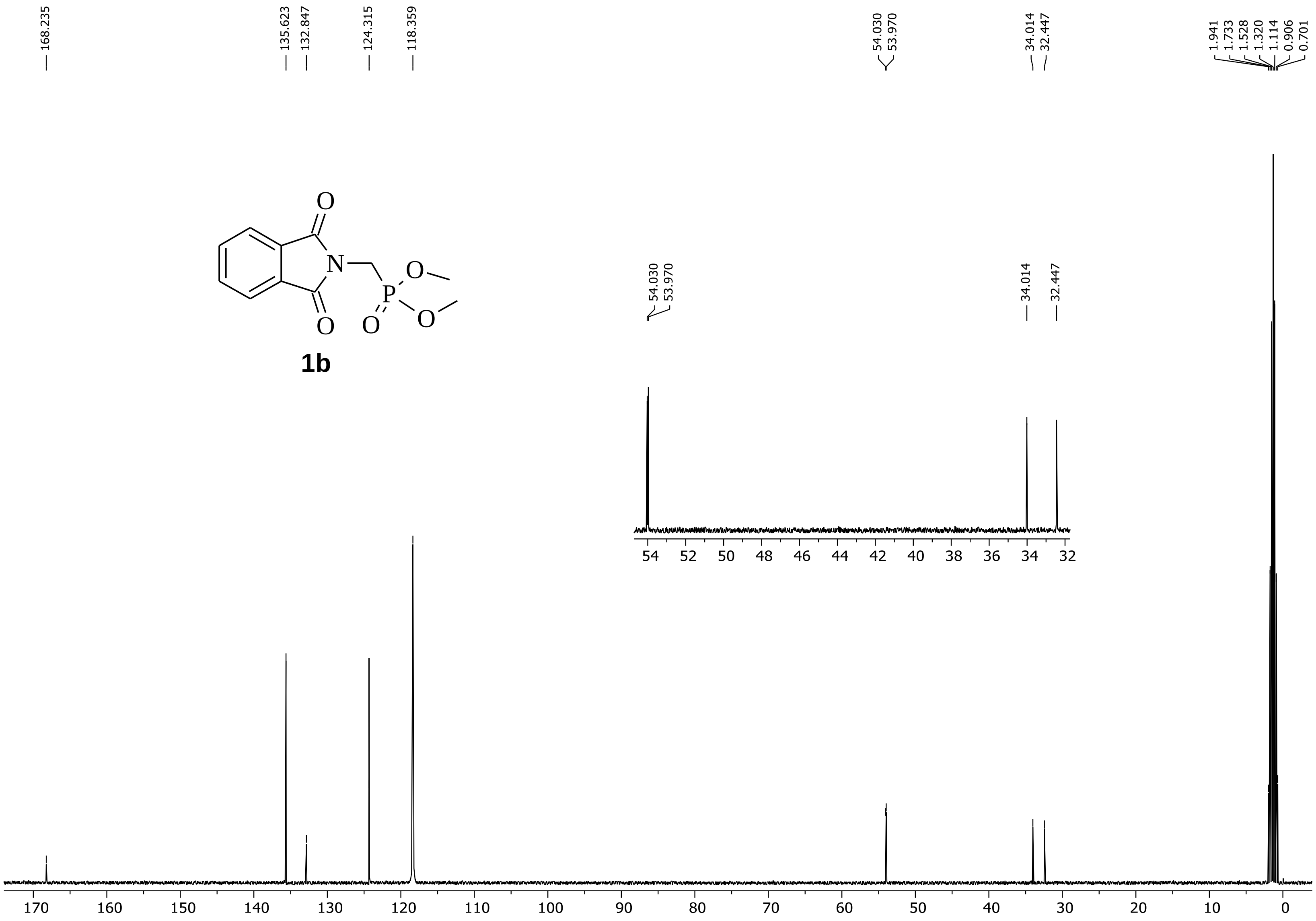

^{13}C-NMR spectrum of dimethyl *N*-phthalimidomethylphosphonate (**1b**); 100 MHz/CD_3CN; δ (ppm).

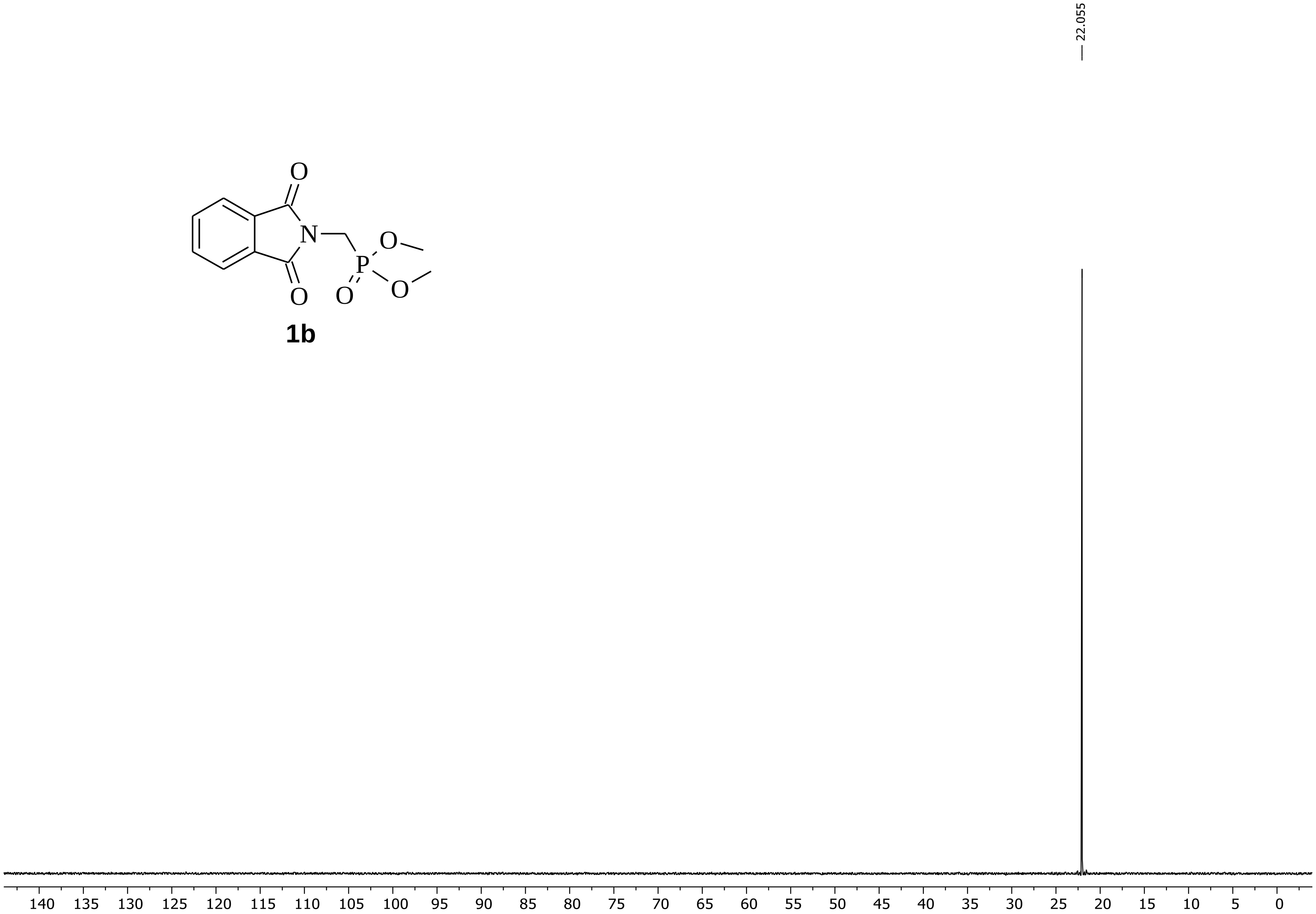

^{31}P-NMR spectrum of dimethyl *N*-phthalimidomethylphosphonate (**1b**); 161.9 MHz/CD_3CN; δ (ppm).

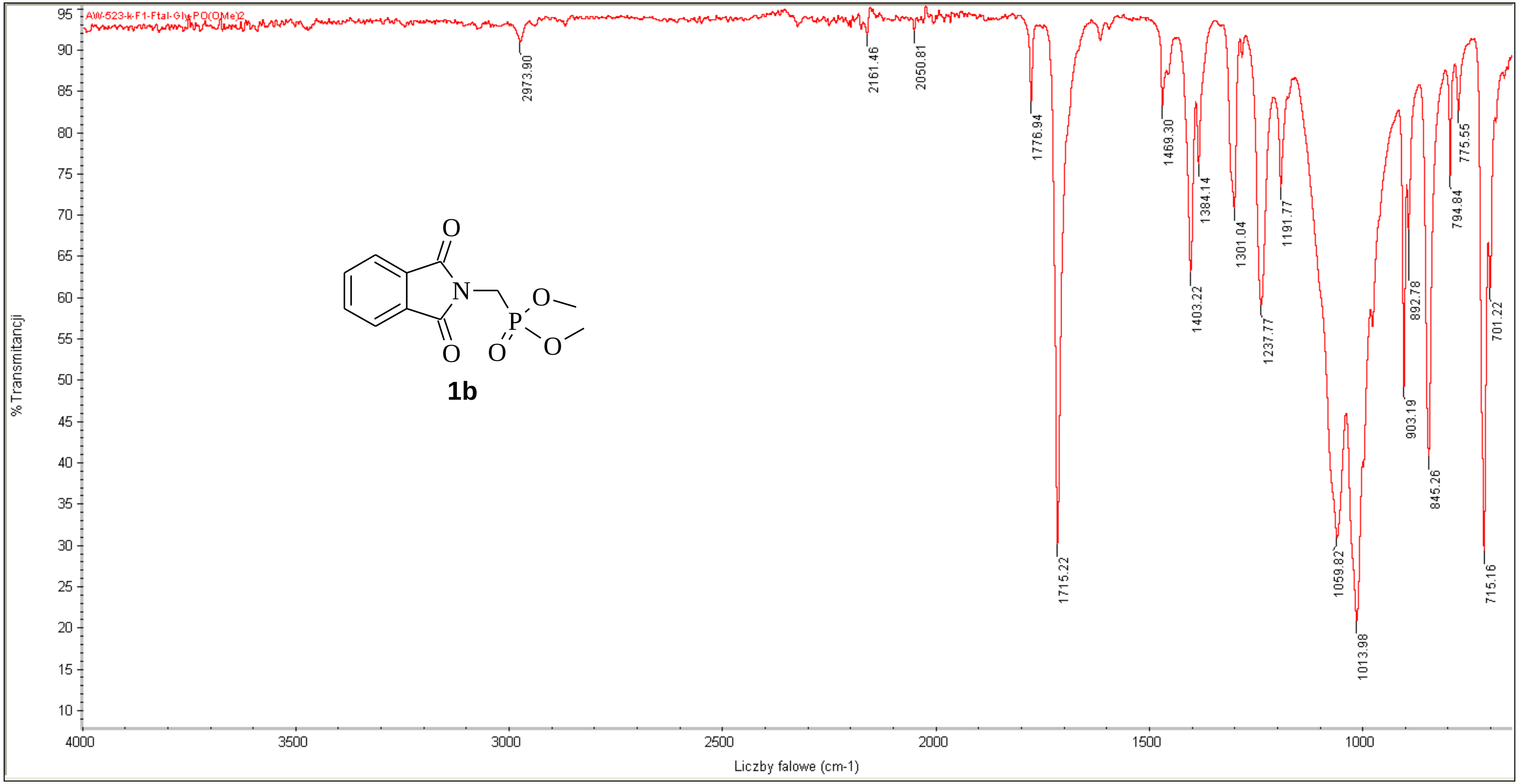

IR spectrum of dimethyl *N*-phthalimidomethylphosphonate (**1b**); ATR, cm^{-1}

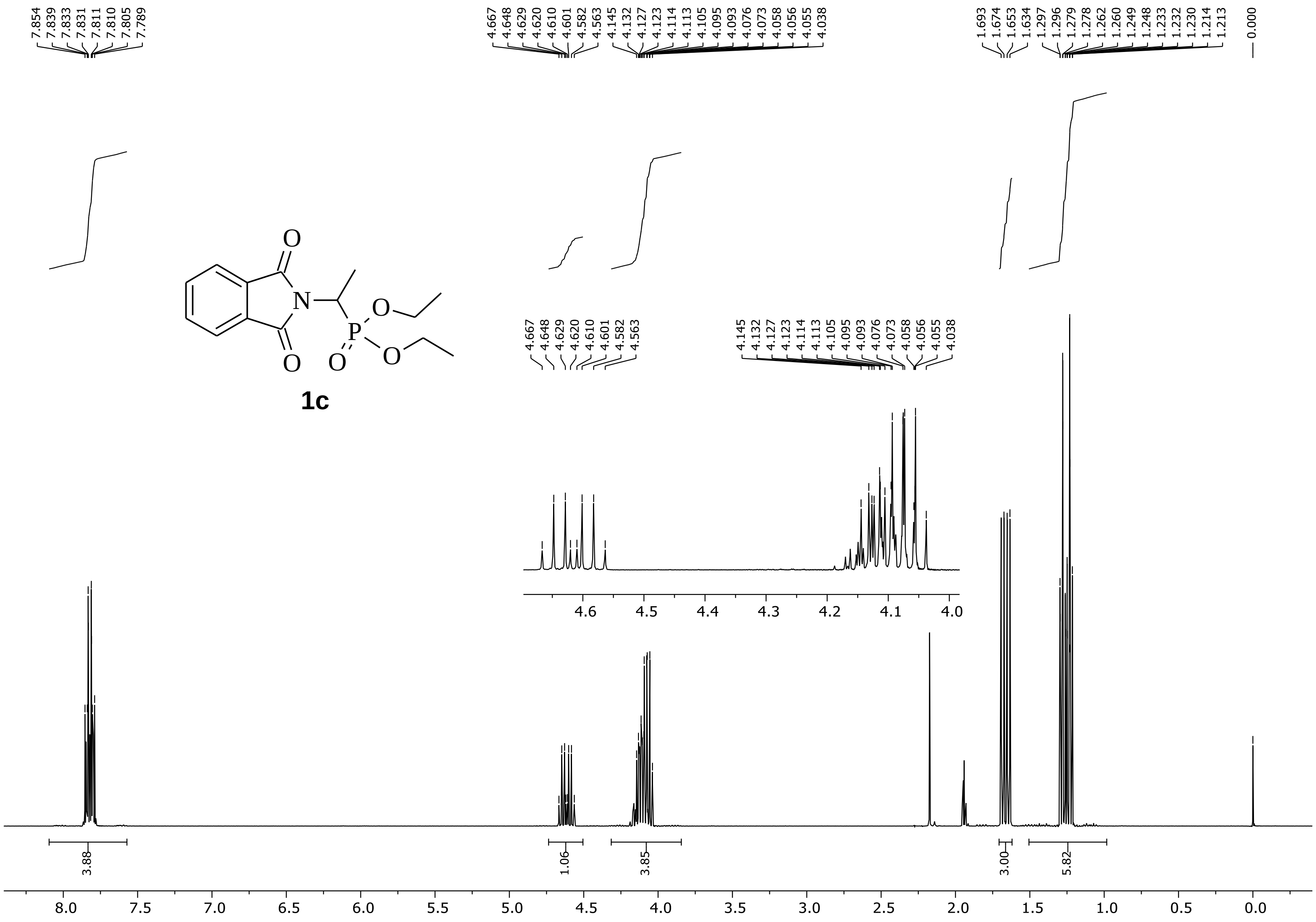

^{1}H-NMR spectrum of diethyl 1-(*N*-phthalimido)ethylphosphonate (**1c**); 400 MHz/CD_3CN/TMS; δ (ppm).

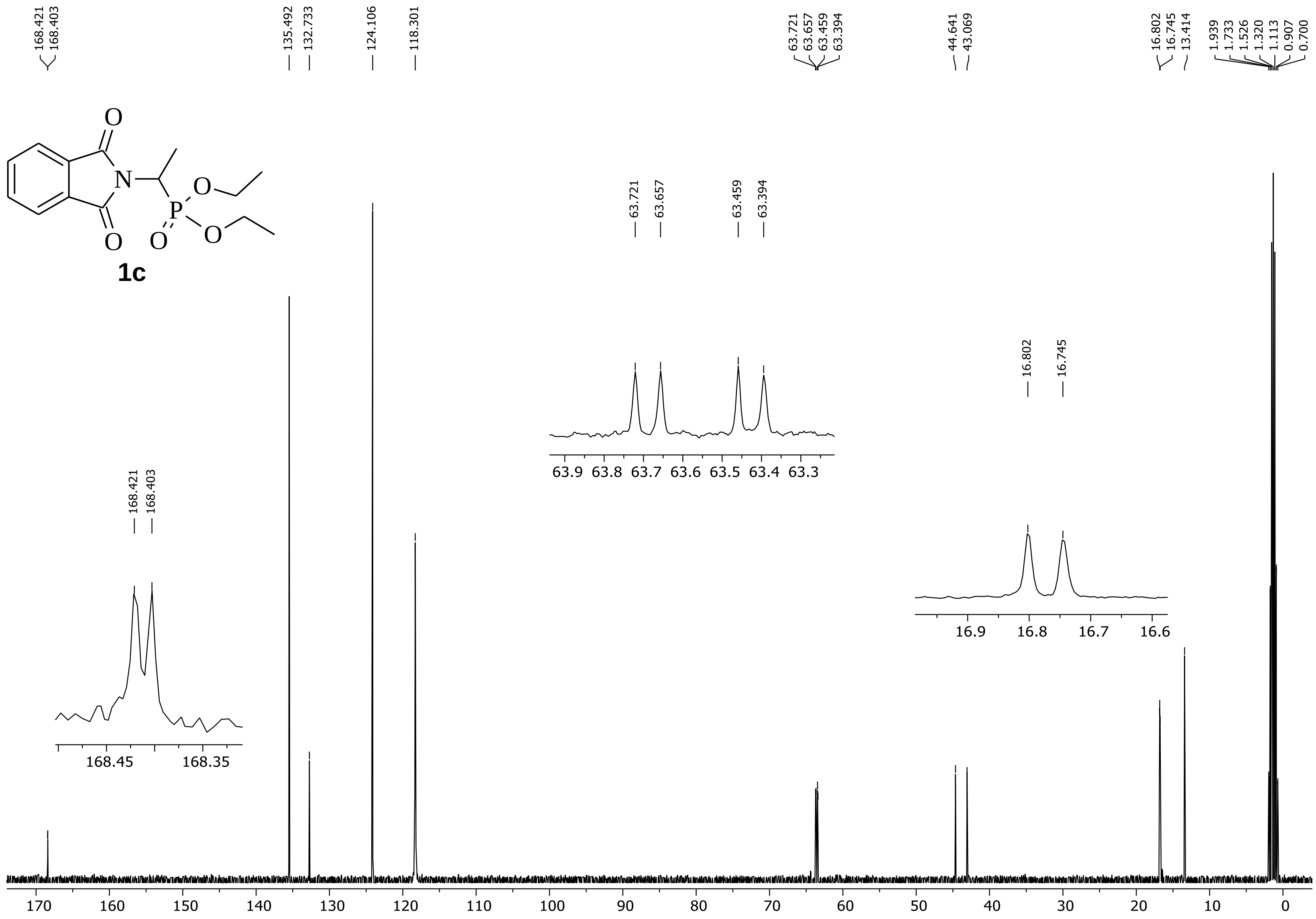

^{13}C-NMR spectrum of diethyl 1-(*N*-phthalimido)ethylphosphonate (**1c**); 100 MHz/CD_3CN; δ (ppm).

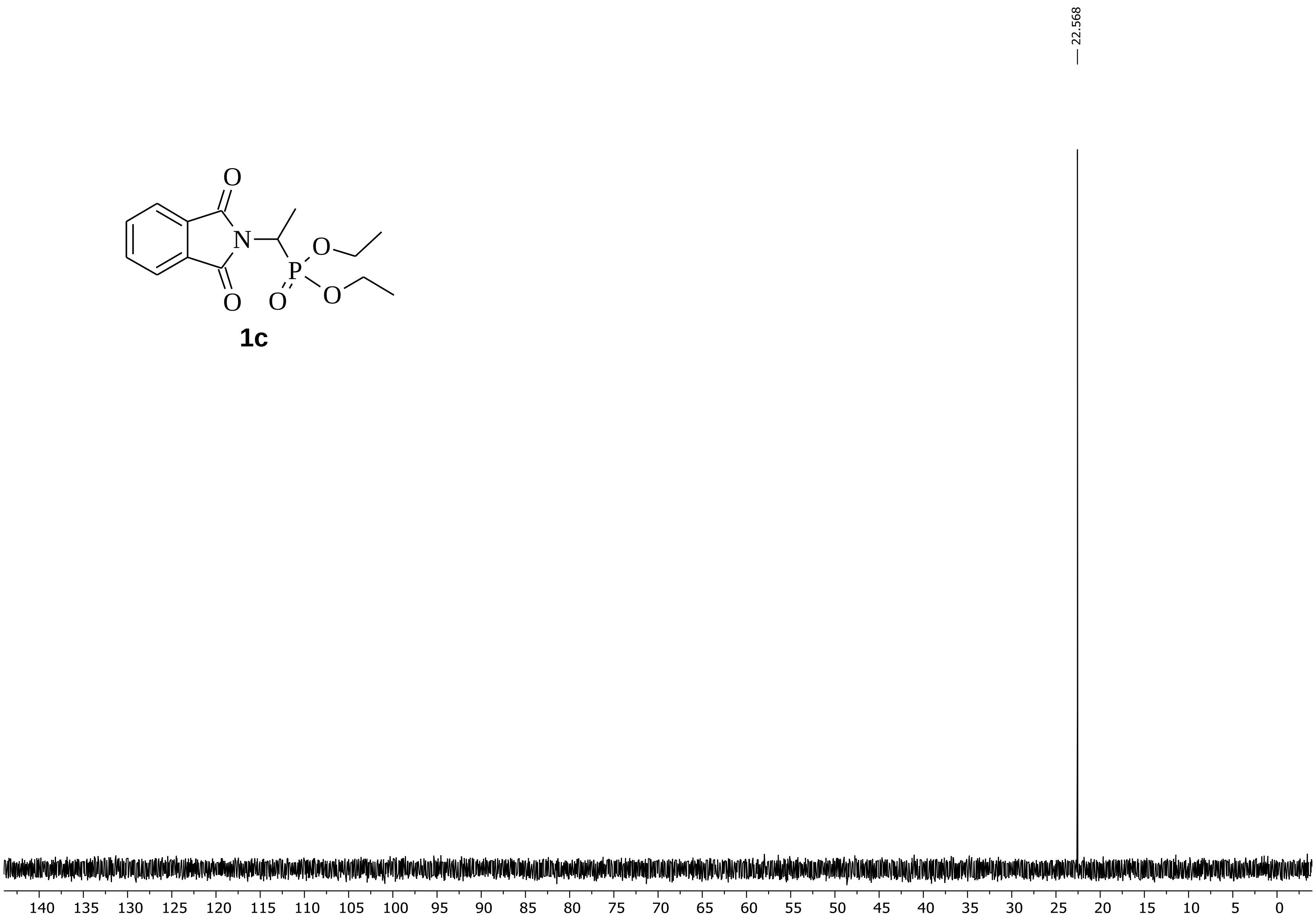

1c

^{31}P-NMR spectrum of diethyl 1-(*N*-phthalimido)ethylphosphonate (**1c**); 161.9 MHz/CD_3CN; δ (ppm).

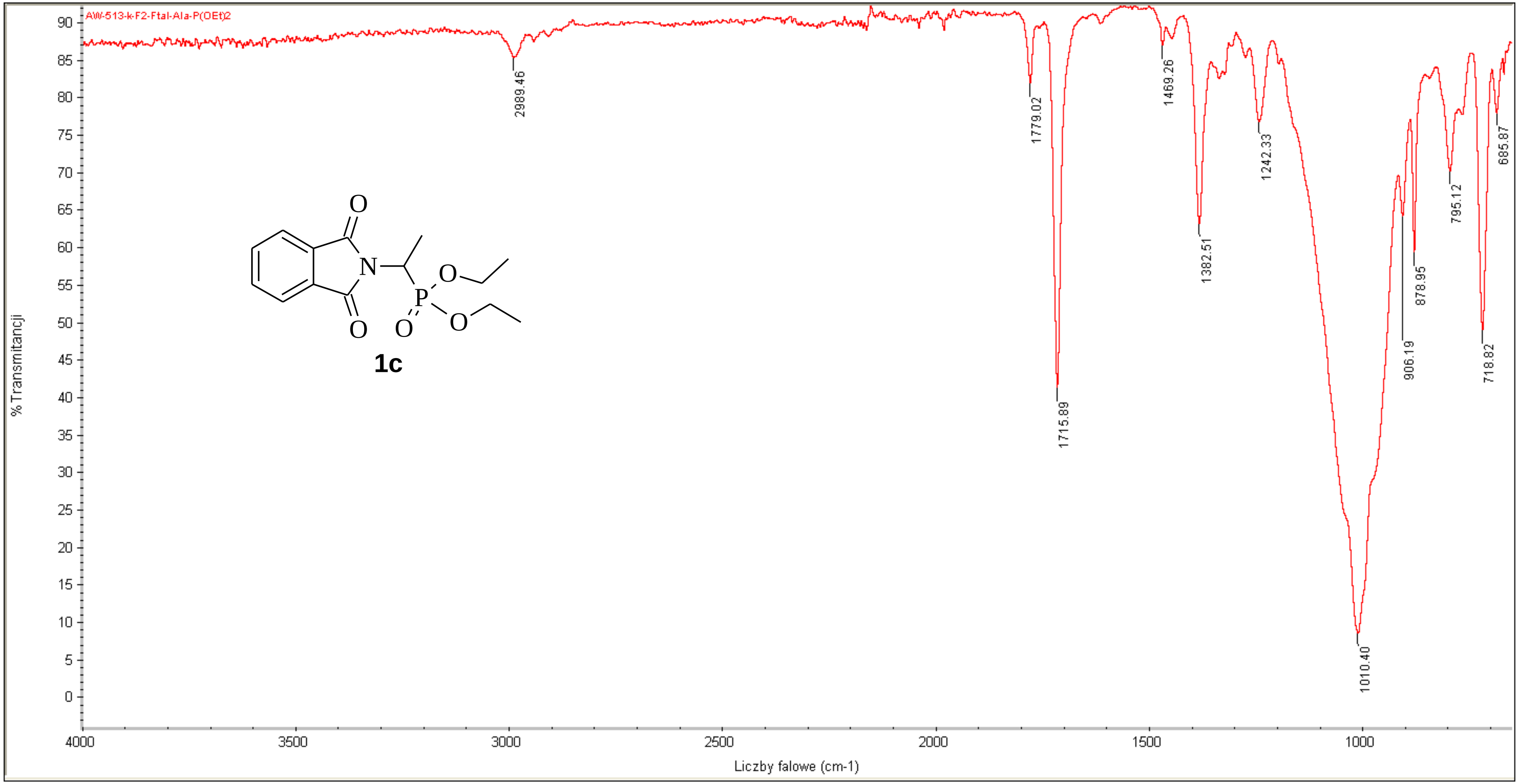

IR spectrum of diethyl 1-(*N*-phthalimido)ethylphosphonate (**1c**); ATR, cm^{-1}.

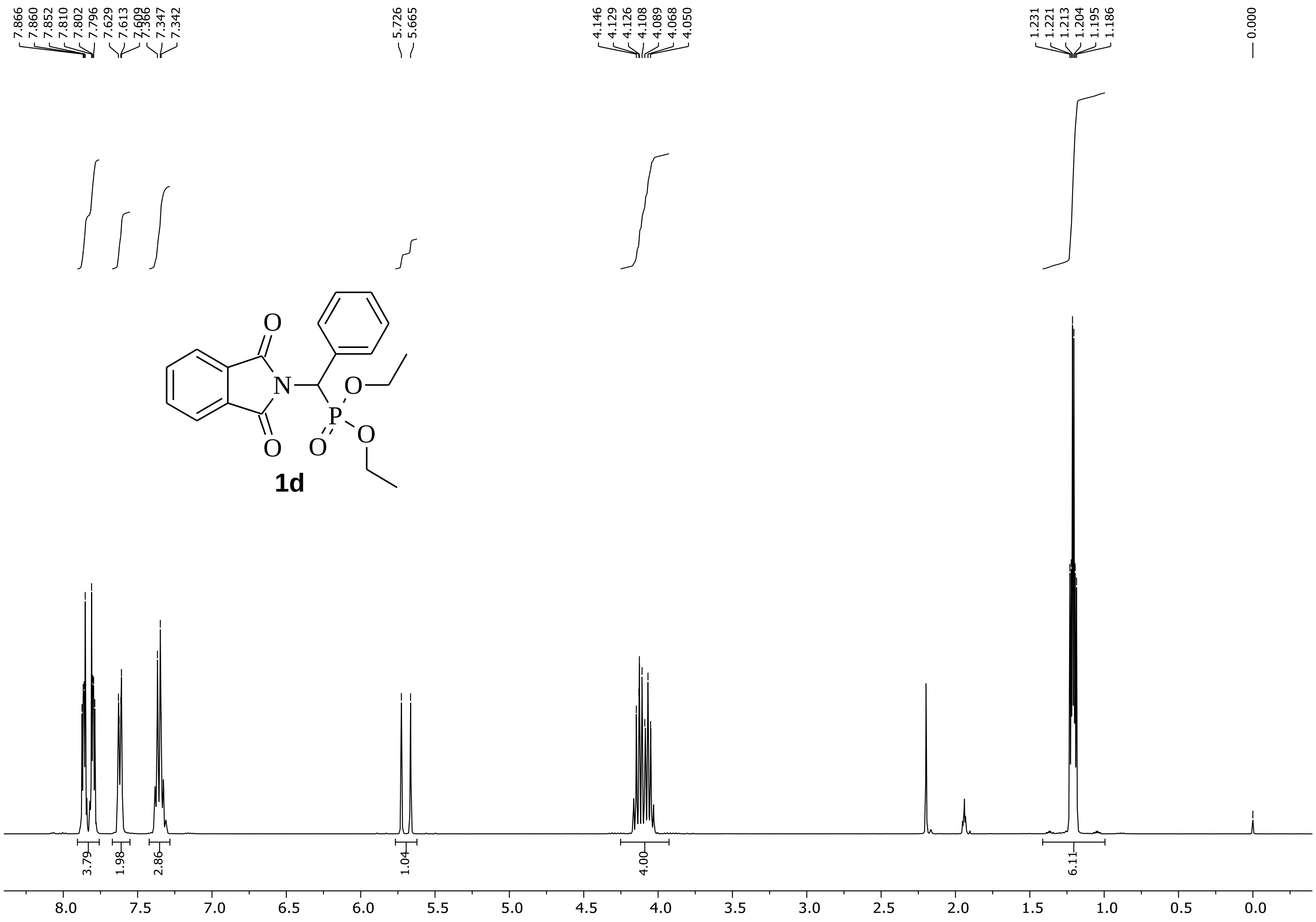

^{1}H-NMR spectrum of diethyl phenyl(*N*-phthalimido)methylphosphonate (**1d**); 400 MHz/CD_3CN/TMS; δ (ppm).

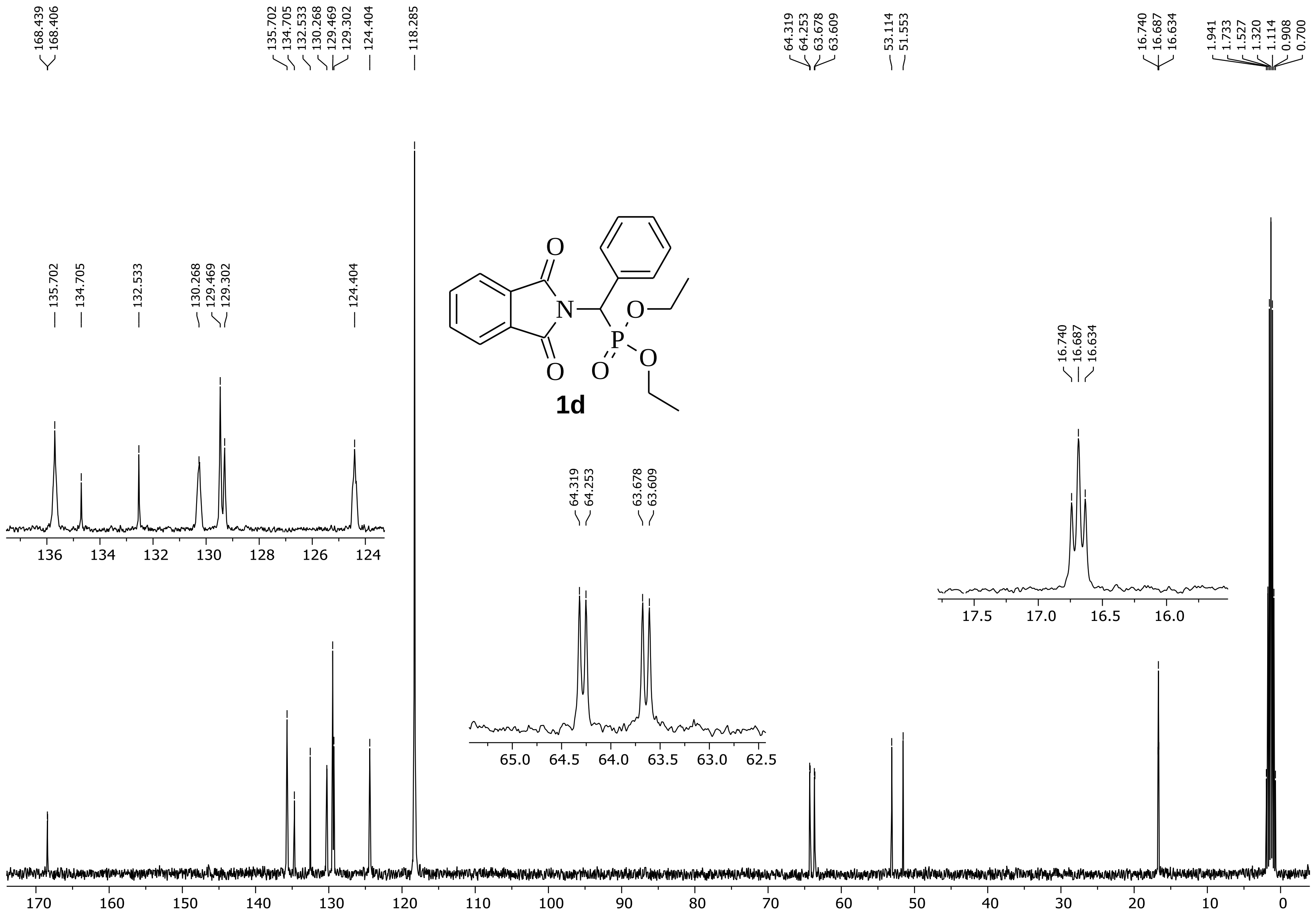

^{13}C-NMR spectrum of diethyl phenyl(*N*-phthalimido)methylphosphonate (**1d**); 100 MHz/CD_3CN; δ (ppm).

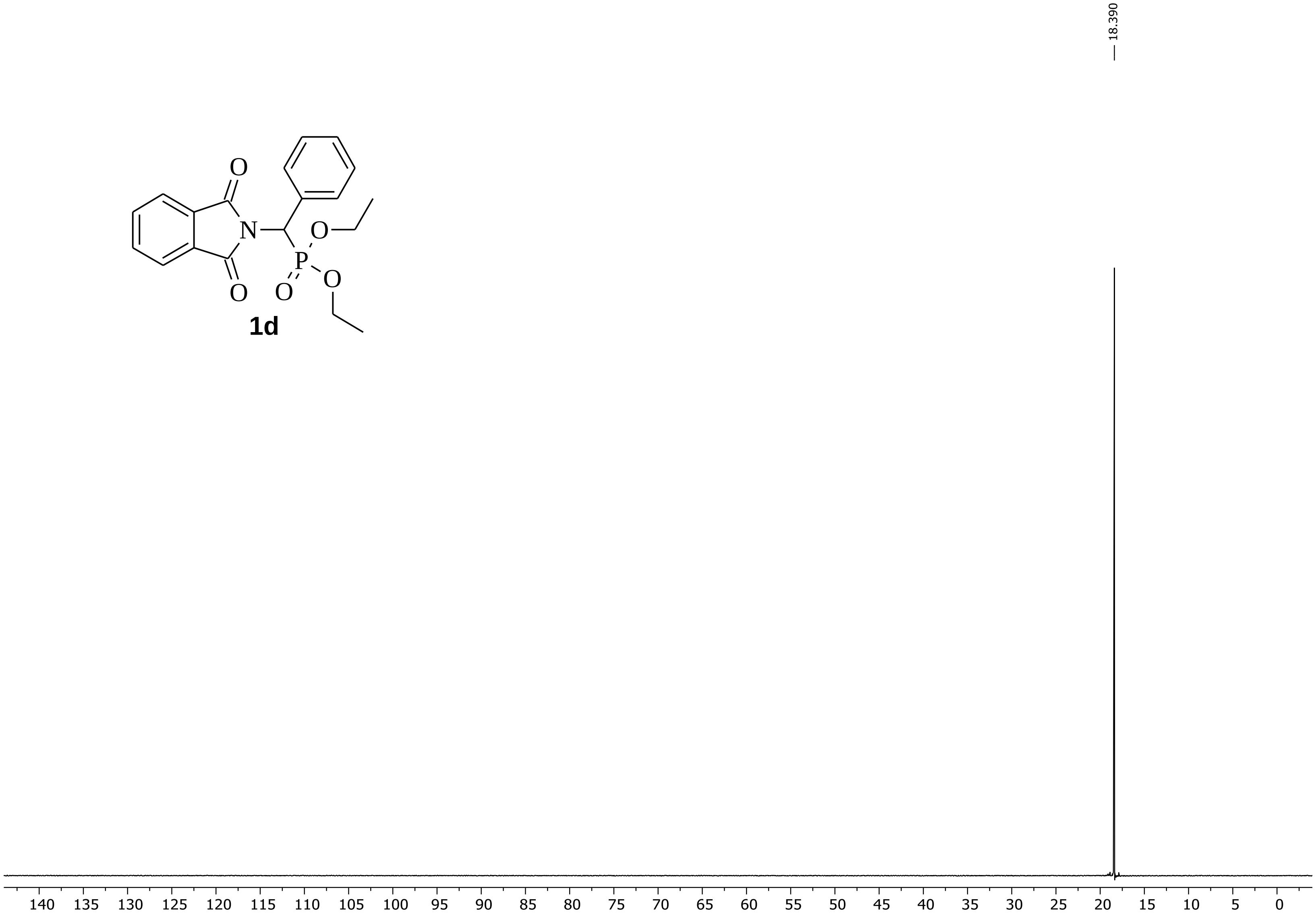

^{31}P-NMR spectrum of diethyl phenyl(*N*-phthalimido)methylphosphonate (**1d**); 161.9 MHz/CD_3CN; δ (ppm).

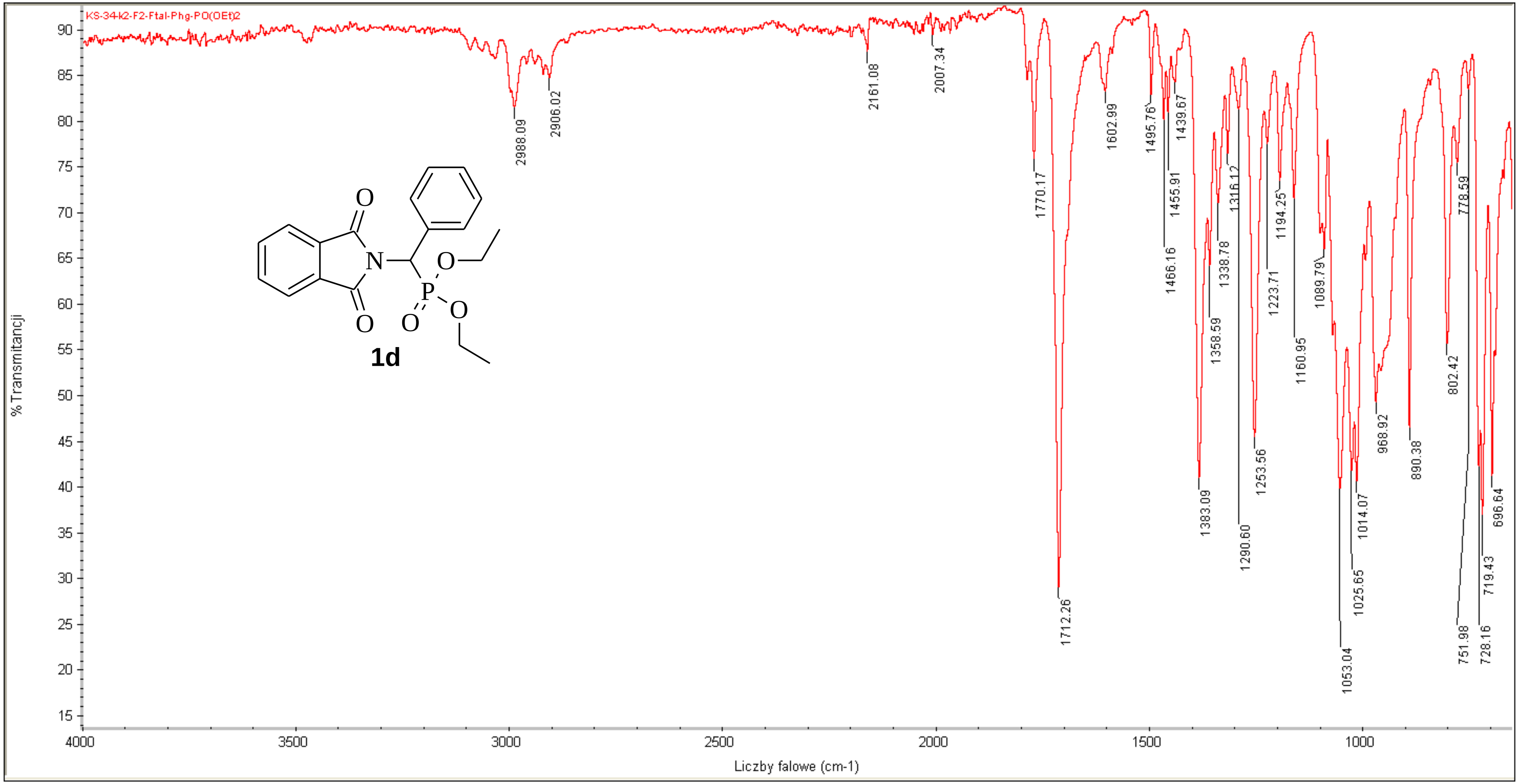

IR spectrum of diethyl phenyl(*N*-phthalimido)methylphosphonate (**1d**); ATR, cm^{-1}.

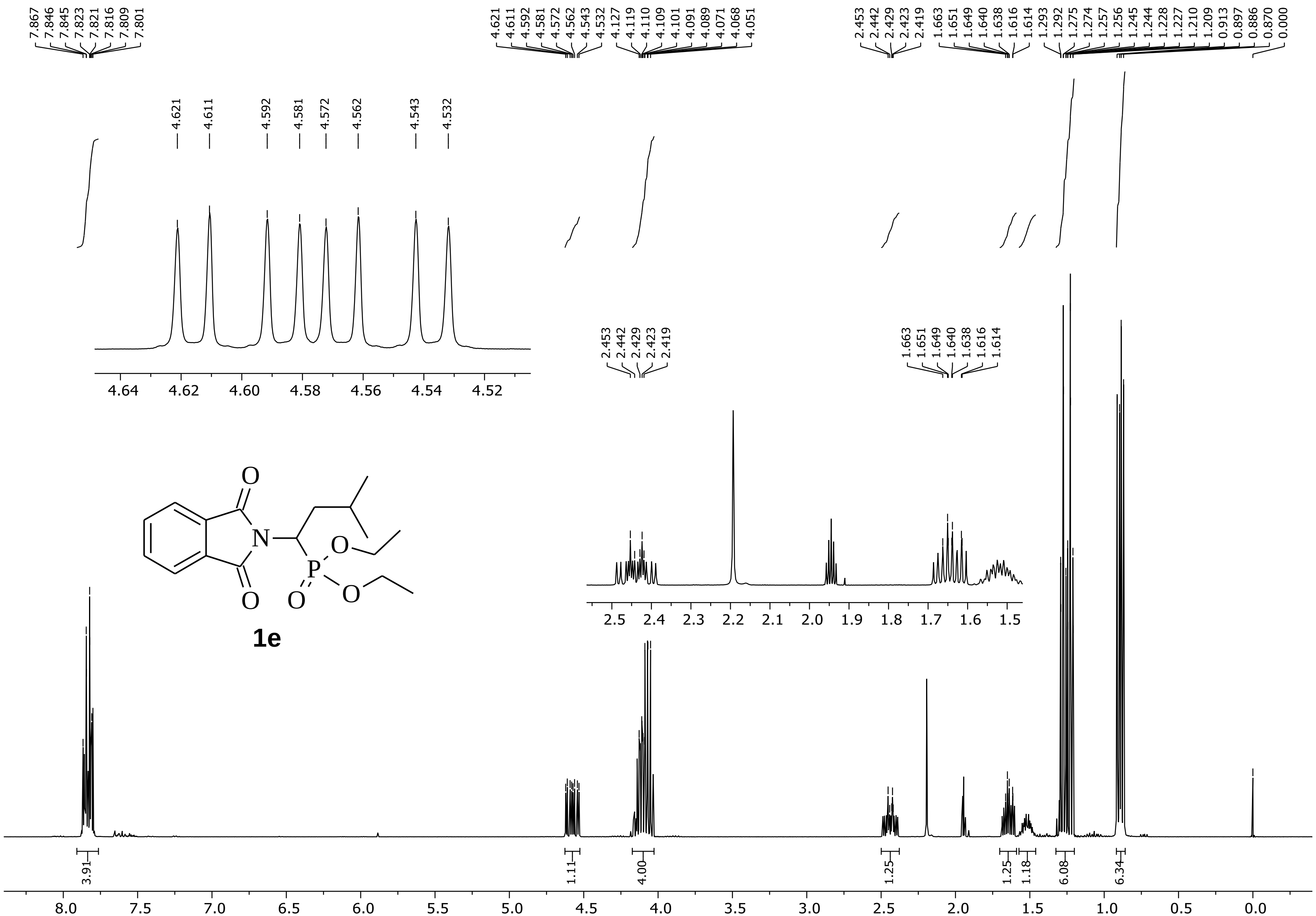

1e

^{1}H-NMR spectrum of diethyl 3-methyl-1-(*N*-phthalimido)butylphosphonate (**1e**); 400 MHz/CD_3CN/TMS; δ (ppm).

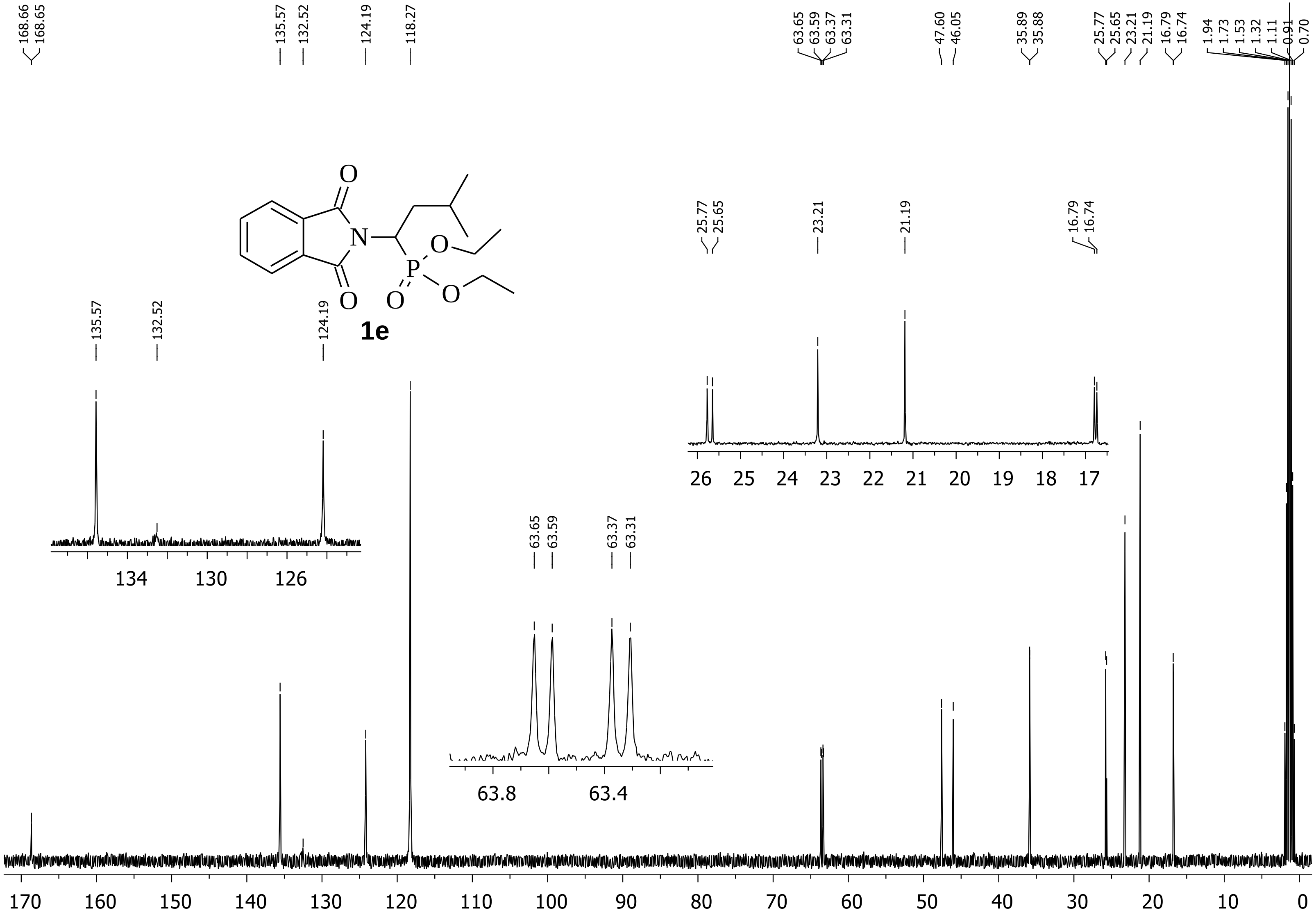

^{13}C-NMR spectrum of diethyl 3-methyl-1-(*N*-phthalimido)butylphosphonate (**1e**); 100 MHz/CD_3CN; δ (ppm).

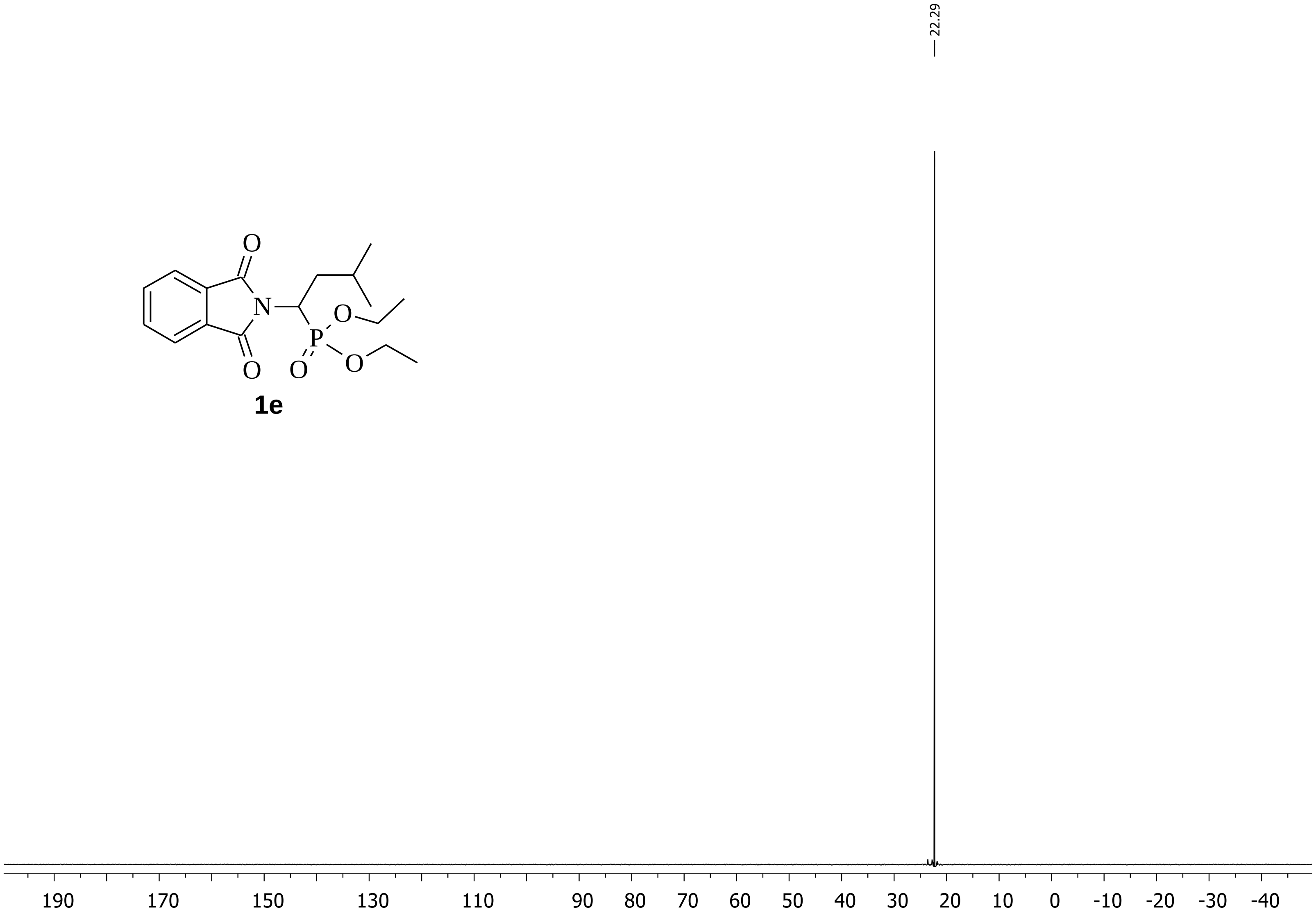

^{31}P-NMR spectrum of diethyl 3-methyl-1-(*N*-phthalimido)butylphosphonate (**1e**); 161.9 MHz/CD_3CN; δ (ppm).

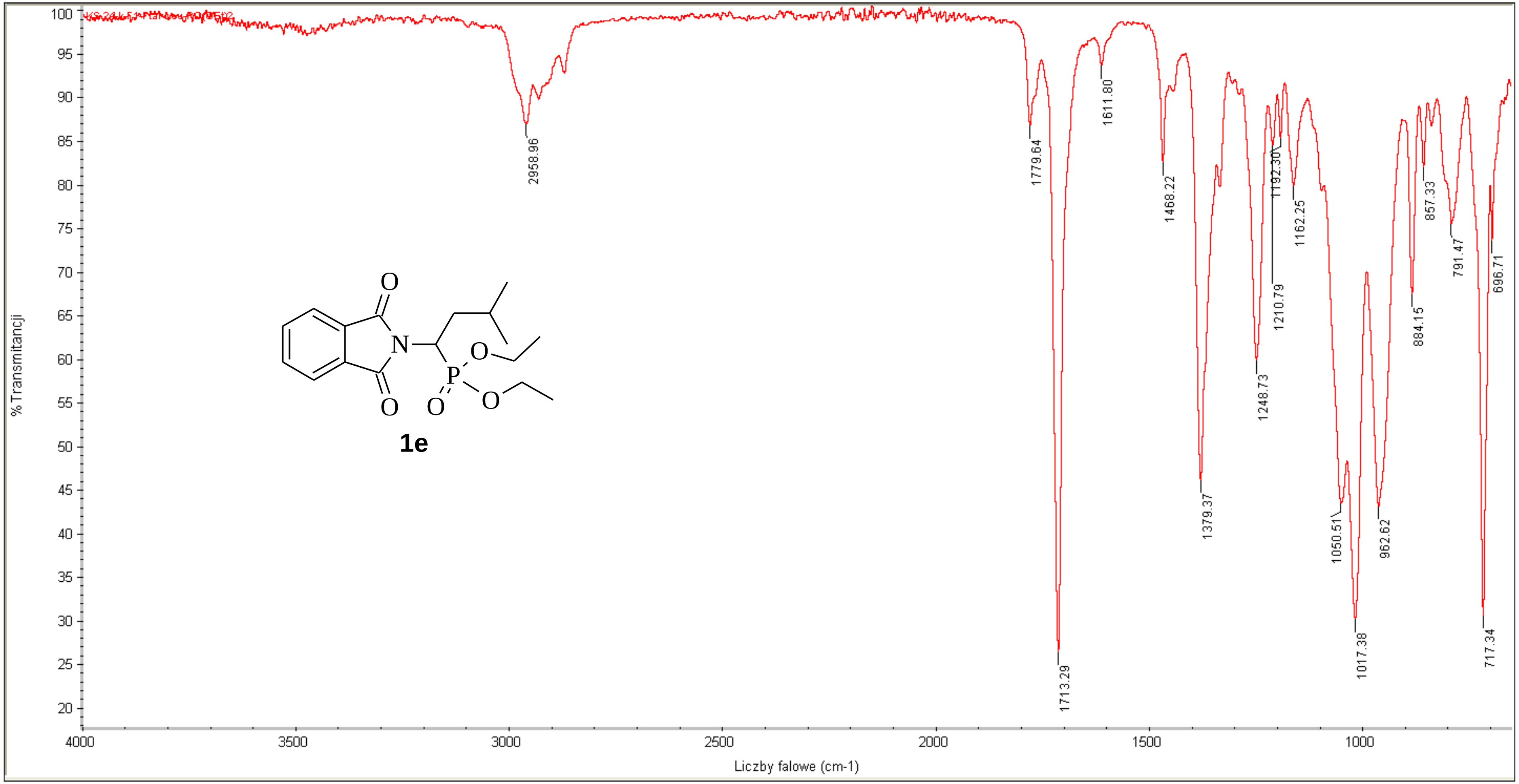

IR spectrum of diethyl 3-methyl-1-(*N*-phthalimido)butylphosphonate (**1e**); ATR, cm^{-1}.

Tolerance = 100.0 mDa / DBE: min = -10.0, max = 200.0
Element prediction: Off
Number of isotope peaks used for i-FIT = 3
Monoisotopic Mass, Even Electron Ions
15 formula(e) evaluated with 7 results within limits (all results (up to 1000) for each mass)
Elements Used:

Mass	RA	Calc. Mass	mDa	PPM	DBE	Formula	i-FIT	i-FIT Norm	Fit Conf %	C	H	N	O	P
354.1469	100.00	354.1470	-0.1	-0.3	6.5	C17 H25 N O5 P	665.4	0.105	90.04	17	25	1	5	1
		354.1259	21.0	59.3	11.5	C20 H21 N O3 P	671.9	6.607	0.14	20	21	1	3	1
		354.1834	-36.5	-103.1	5.5	C18 H29 N O4 P	669.7	4.462	1.15	18	29	1	4	1
		354.0895	57.4	162.1	12.5	C19 H17 N O4 P	672.0	6.732	0.12	19	17	1	4	1
		354.2198	-72.9	-205.8	4.5	C19 H33 N O3 P	673.1	7.813	0.04	19	33	1	3	1
		354.0531	93.8	264.9	13.5	C18 H13 N O5 P	671.7	6.436	0.16	18	13	1	5	1
		354.2409	-94.0	-265.4	-0.5	C16 H37 N O5 P	667.8	2.483	8.35	16	37	1	5	1

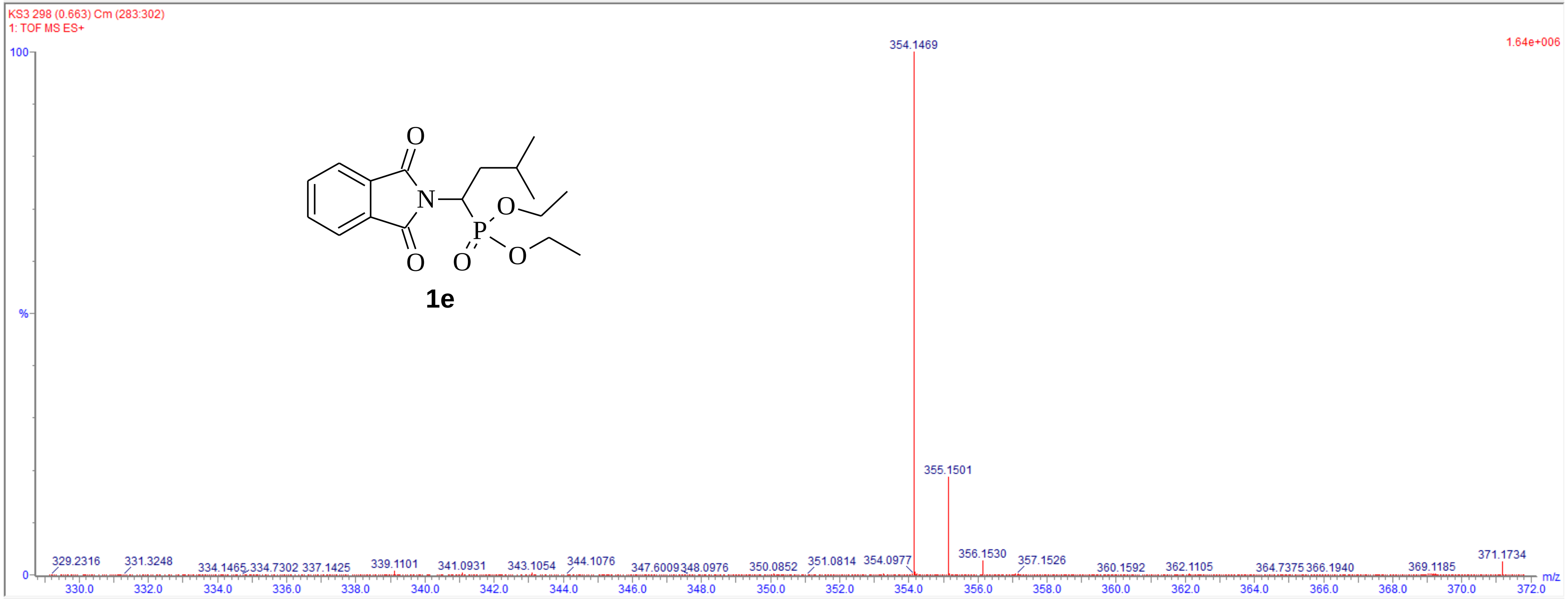

For Help, press F1

HRMS spectrum of diethyl 3-methyl-1-(*N*-phthalimido)butylphosphonate (**1e**).

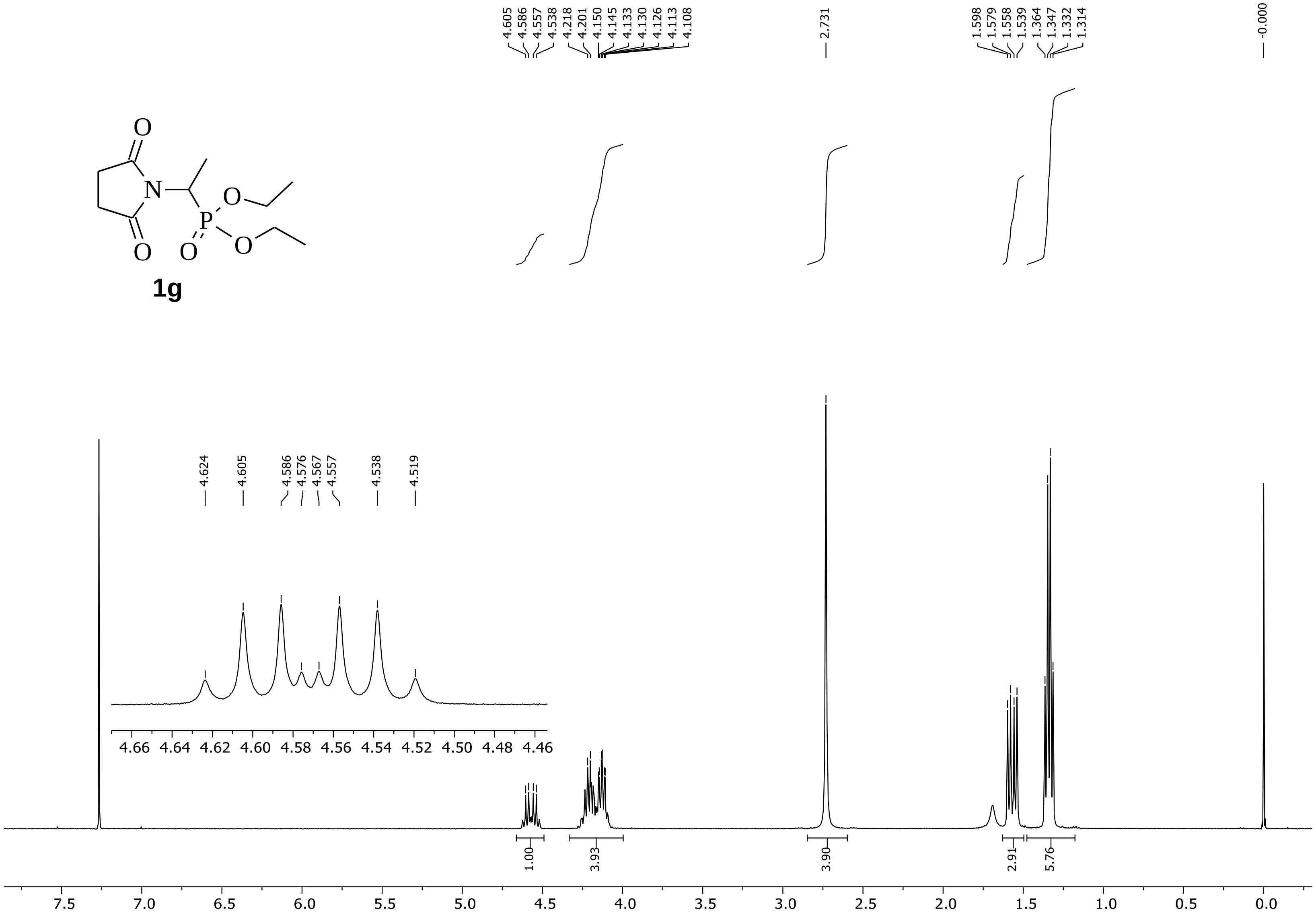

1g

^{1}H-NMR spectrum of diethyl 1-(*N*-succinimido)ethylphosphonate (**1g**); 400 MHz/$CDCl_3$/TMS; δ (ppm).

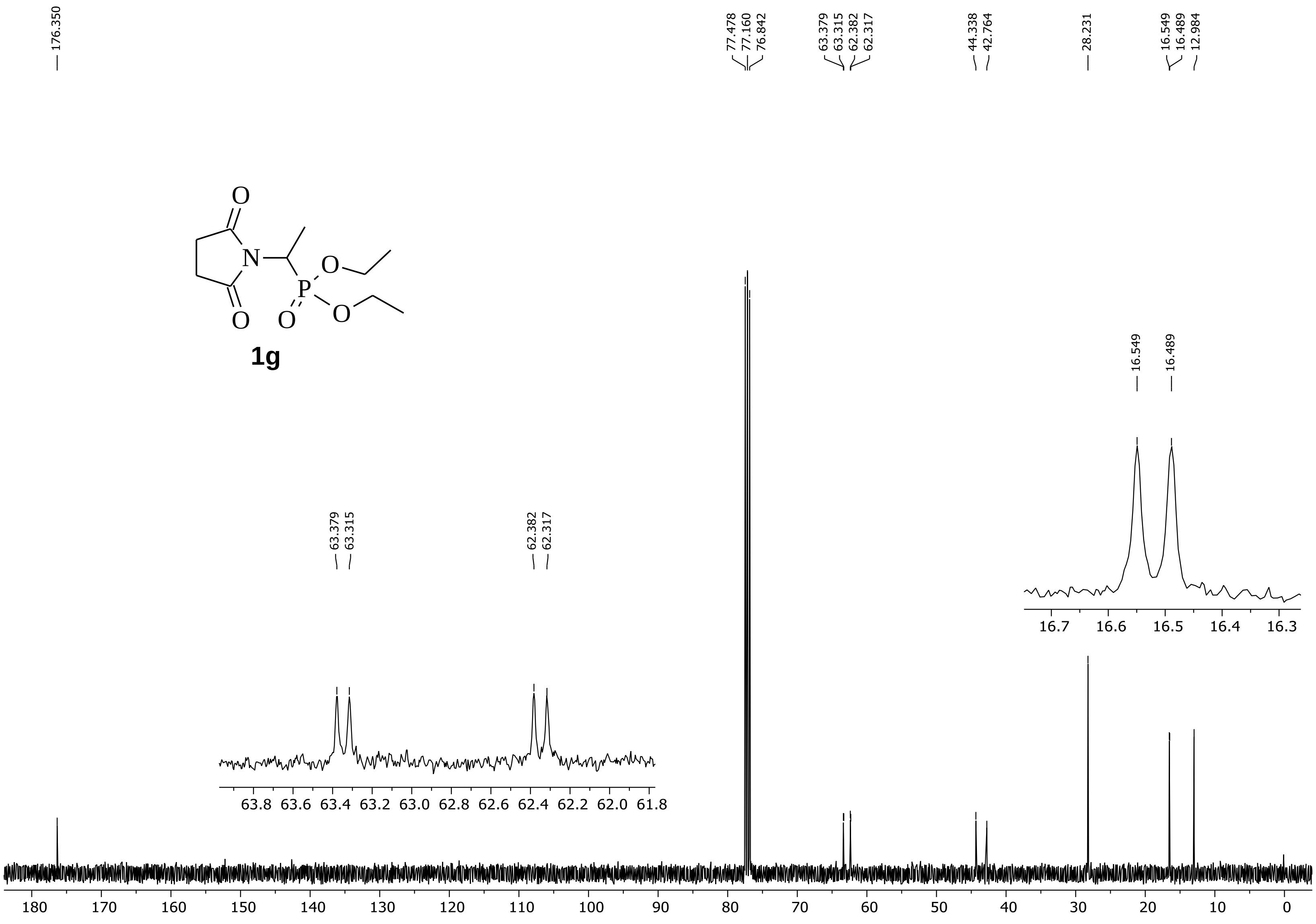

^{13}C-NMR spectrum of diethyl 1-(*N*-succinimido)ethylphosphonate (**1g**); 100 MHz/$CDCl_3$; δ (ppm).

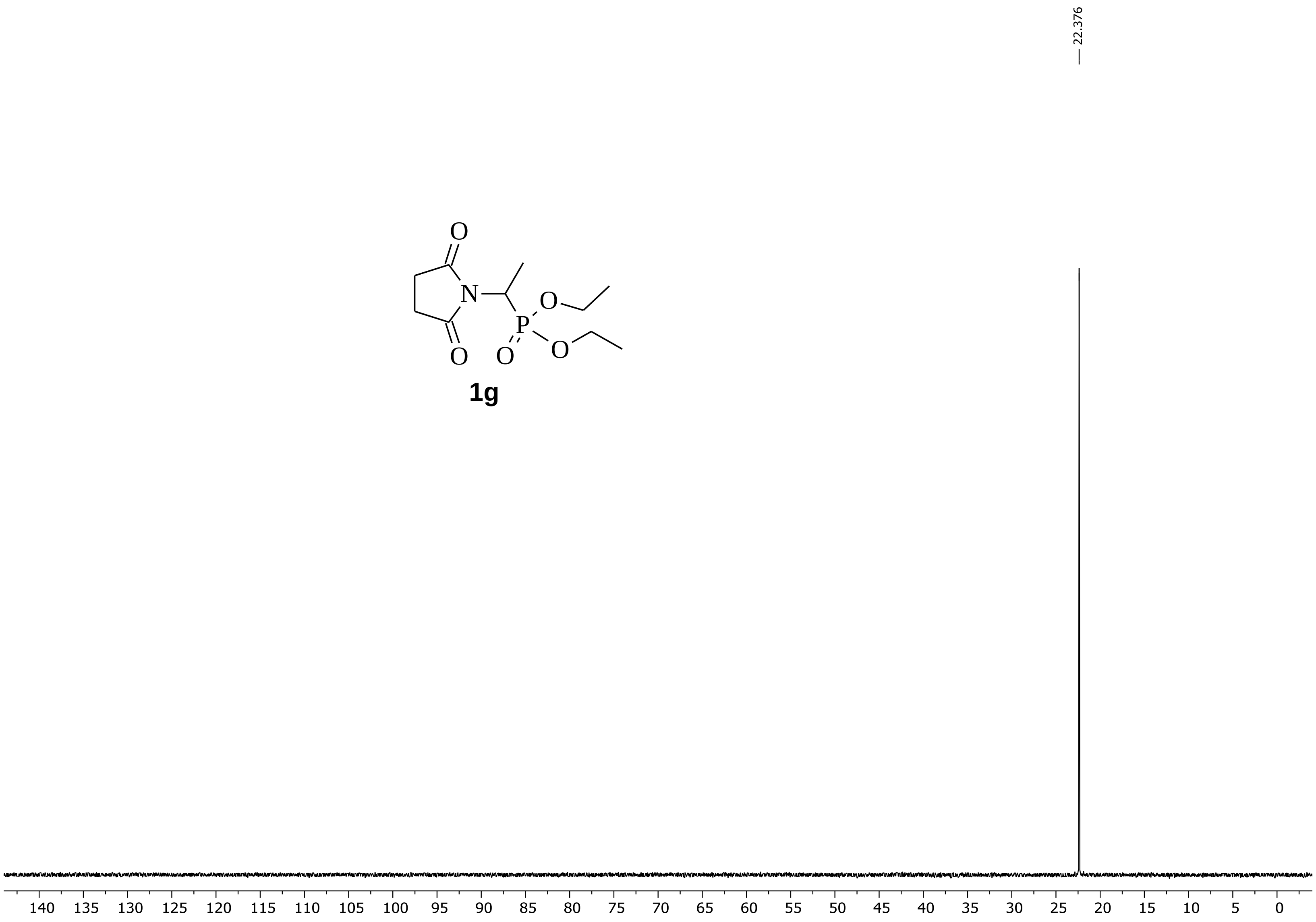

^{31}P-NMR spectrum of diethyl 1-(*N*-succinimido)ethylphosphonate (**1g**); 161.9 MHz/$CDCl_3$; δ (ppm).

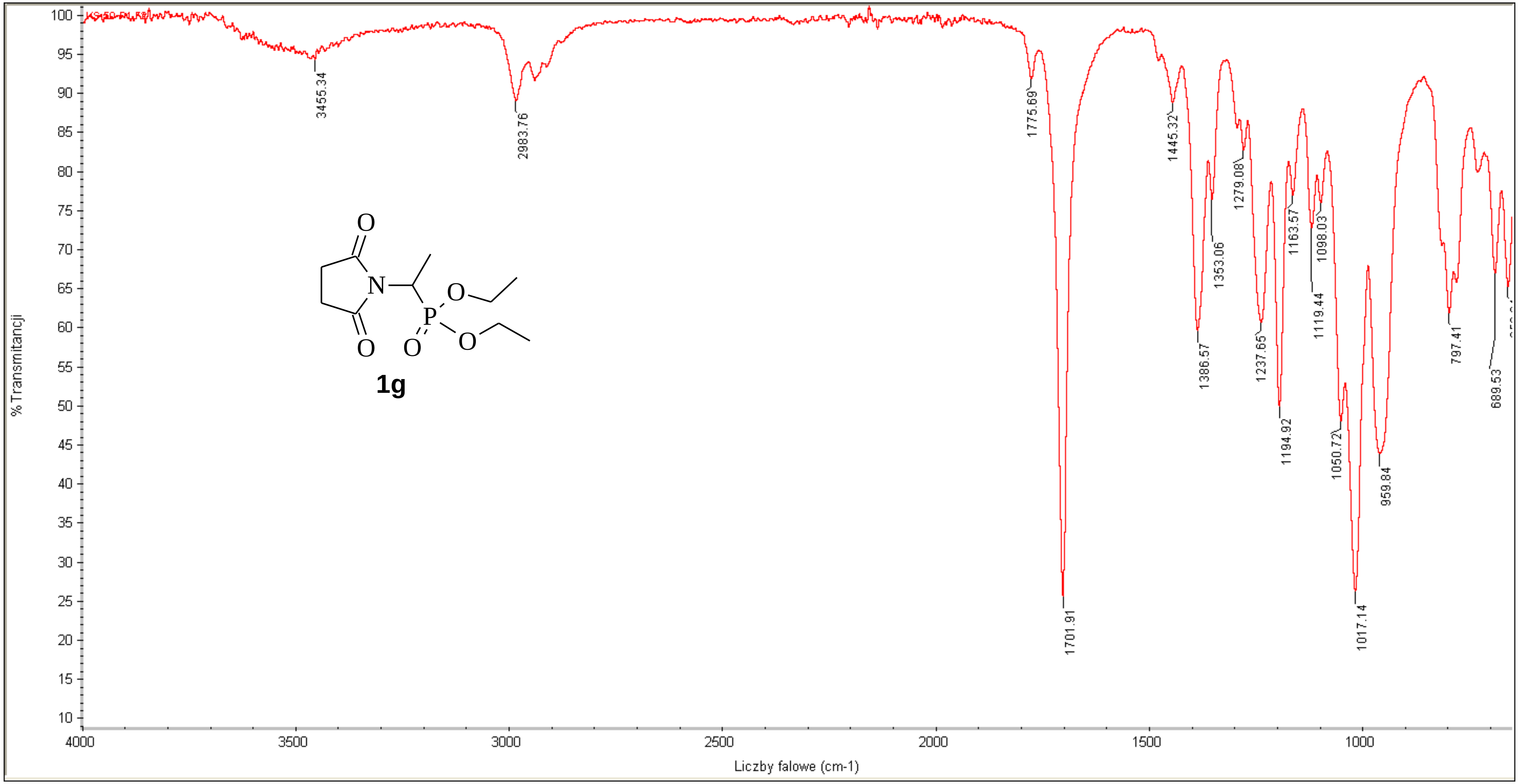

IR spectrum of diethyl 1-(*N*-succinimido)ethylphosphonate (**1g**); ATR, cm^{-1}.

Tolerance = 100.0 mDa / DBE: min = -10.0, max = 200.0
Element prediction: Off
Number of isotope peaks used for i-FIT = 3
Monoisotopic Mass, Even Electron Ions
13 formula(e) evaluated with 7 results within limits (up to 3 closest results for each mass)
Elements Used:

Mass	RA	Calc. Mass	mDa	PPM	DBE	Formula	i-FIT	i-FIT Norm	Fit Conf %	C	H	N	O	P
264.1001	100.00	264.1001	0.0	0.0	2.5	C10 H19 N O5 P	611.7	2.064	12.70	10	19	1	5	1
		264.0790	21.1	79.9	7.5	C13 H15 N O3 P	610.0	0.355	70.12	13	15	1	3	1
		264.1365	-36.4	-137.8	1.5	C11 H23 N O4 P	611.4	1.761	17.18	11	23	1	4	1

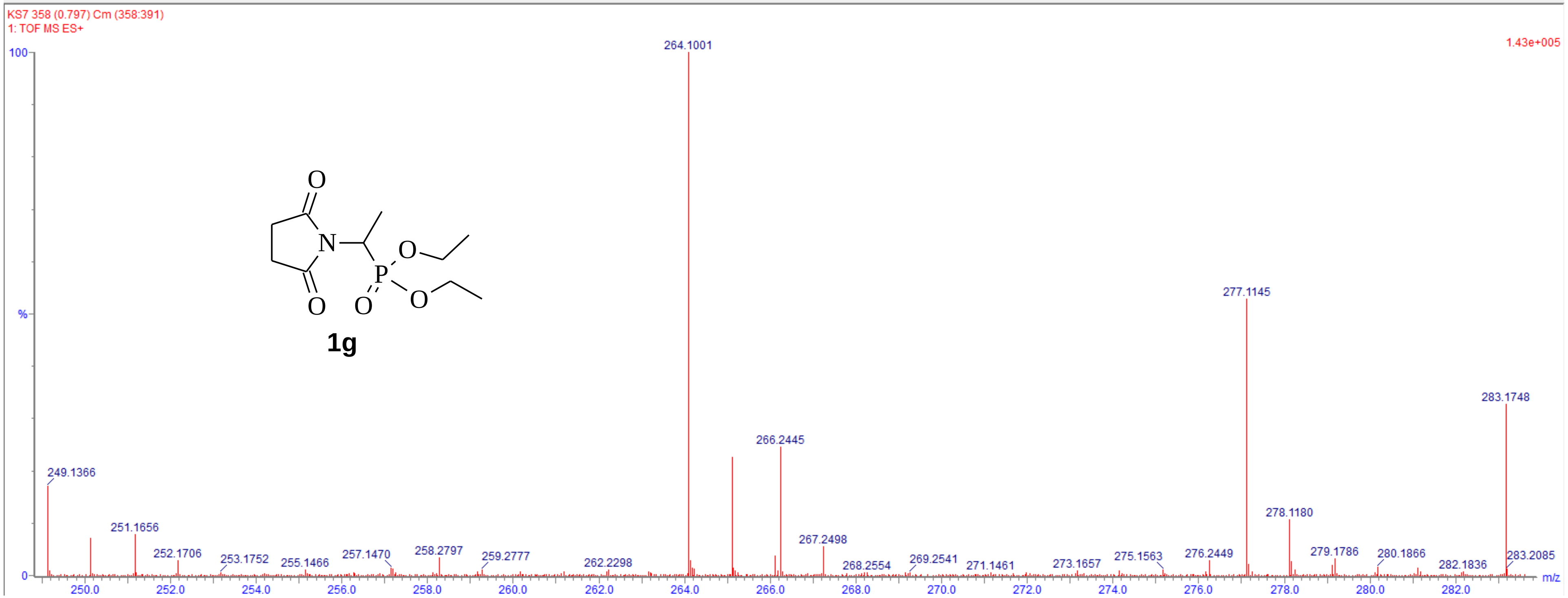

HRMS spectrum of diethyl 1-(*N*-succinimido)ethylphosphonate (**1g**).

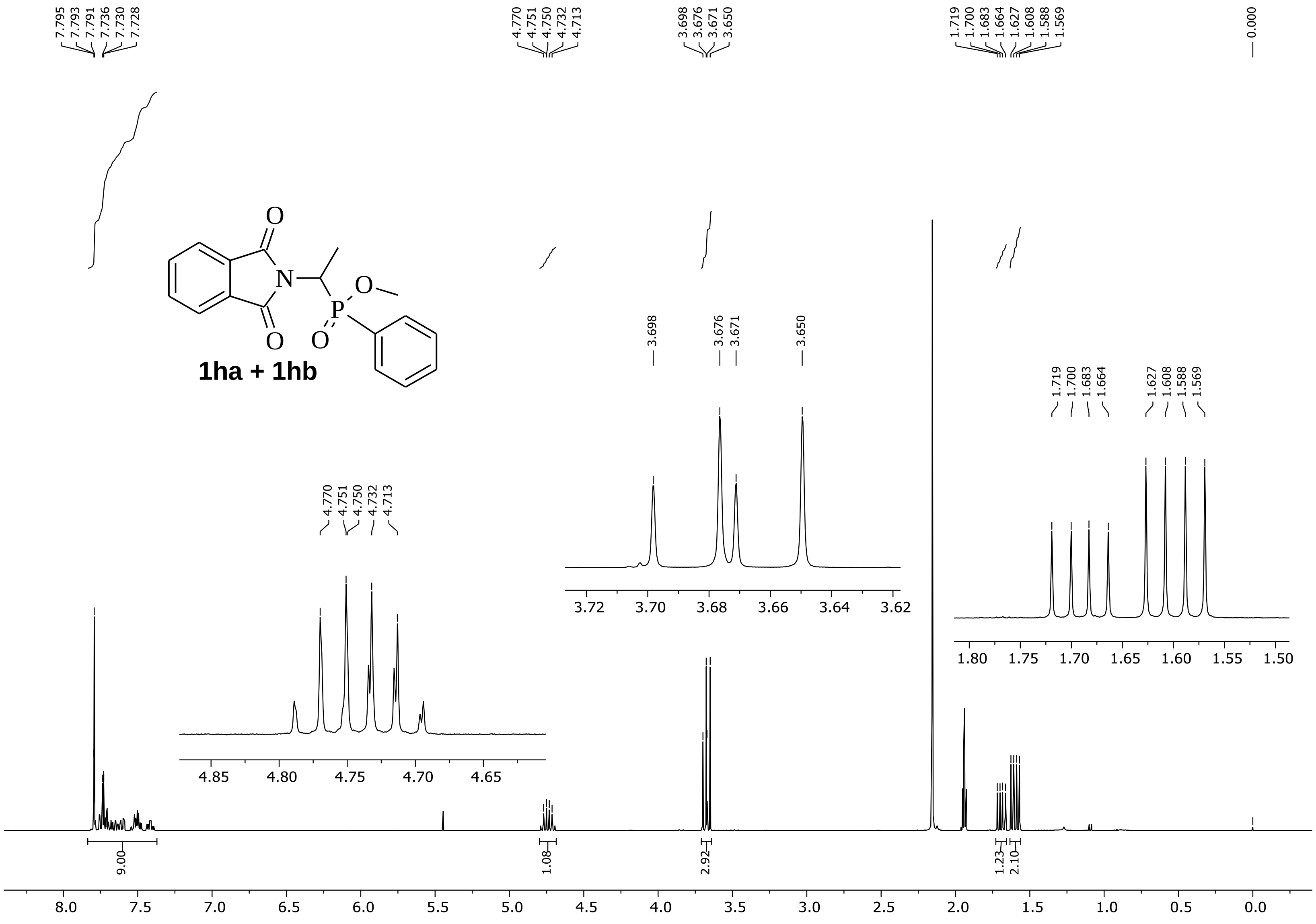

^{1}H-NMR spectrum of methyl phenyl[1-(*N*-phthalimido)ethyl]phosphinate - a mixture of two disatereoisomers (**1ha**+**1hb**); 400 MHz/CD_3CN/TMS; δ (ppm).

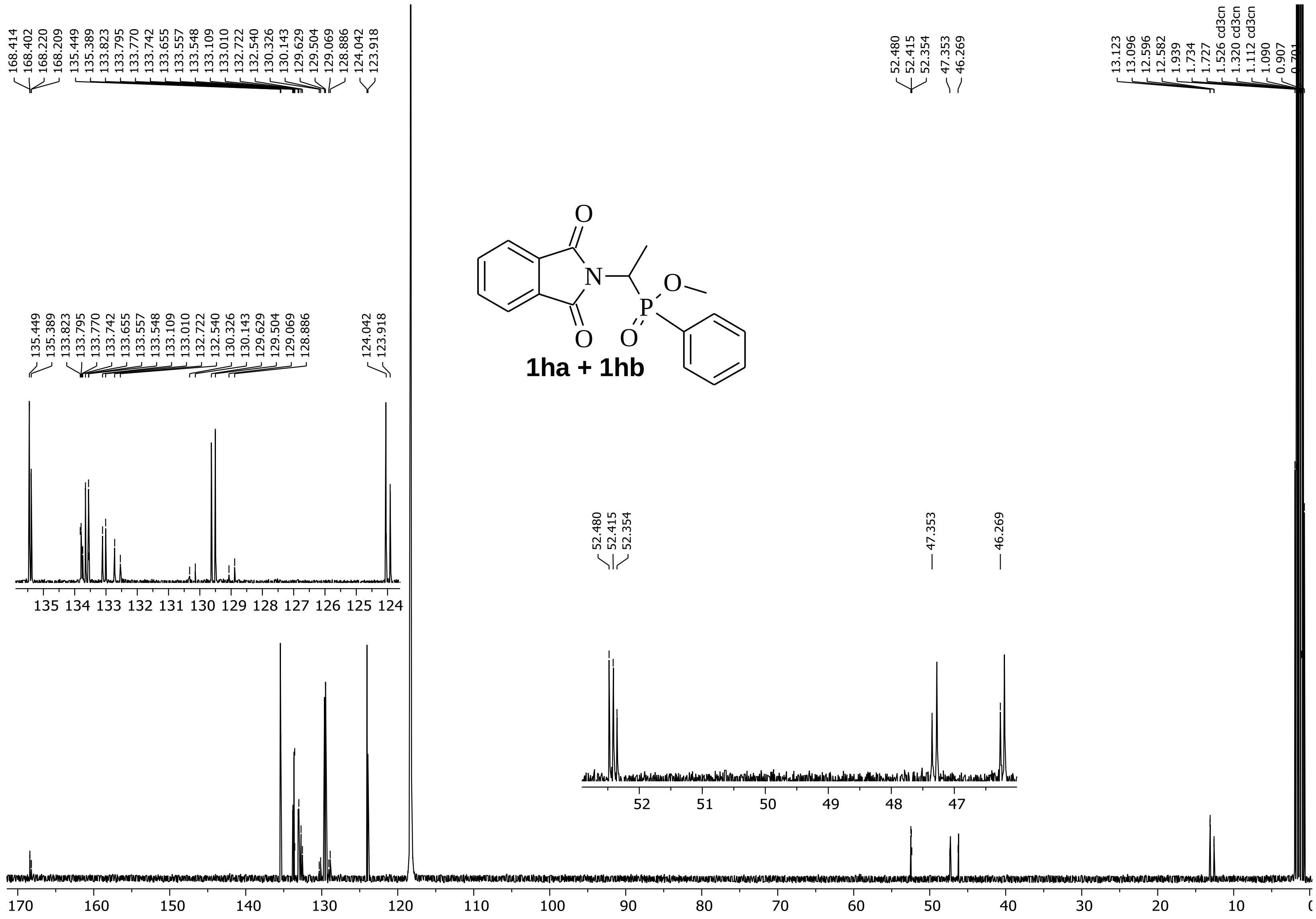

^{13}C-NMR spectrum of methyl phenyl[1-(*N*-phthalimido)ethyl]phosphinate - a mixture of two disatereoisomers (**1ha**+**1hb**); 100 MHz/CD_3CN; δ (ppm).

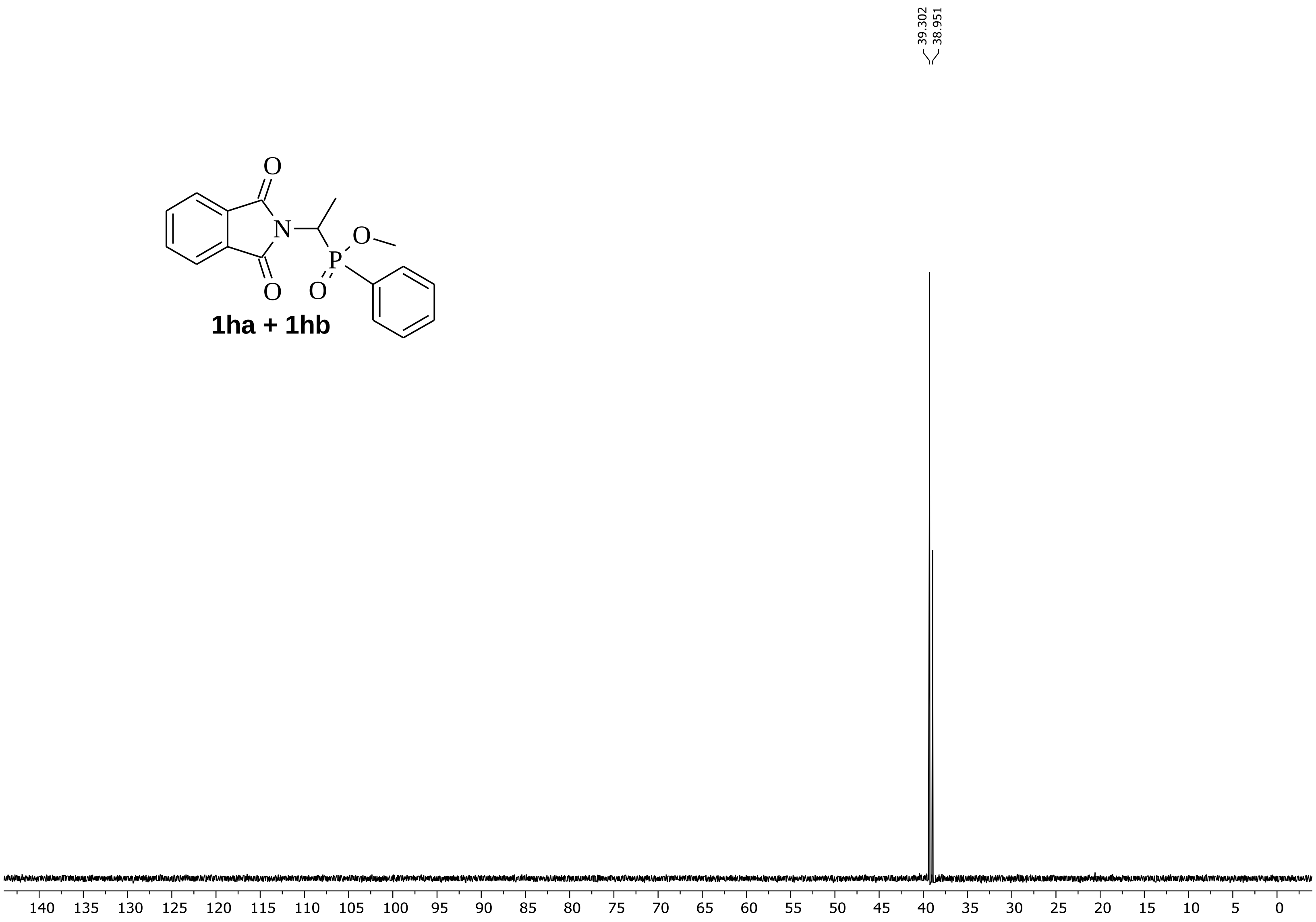

^{31}P-NMR spectrum of methyl phenyl[1-(*N*-phthalimido)ethyl]phosphinate - a mixture of two disatereoisomers (**1ha**+**1hb**); 161.9 MHz/CD_3CN; δ (ppm).

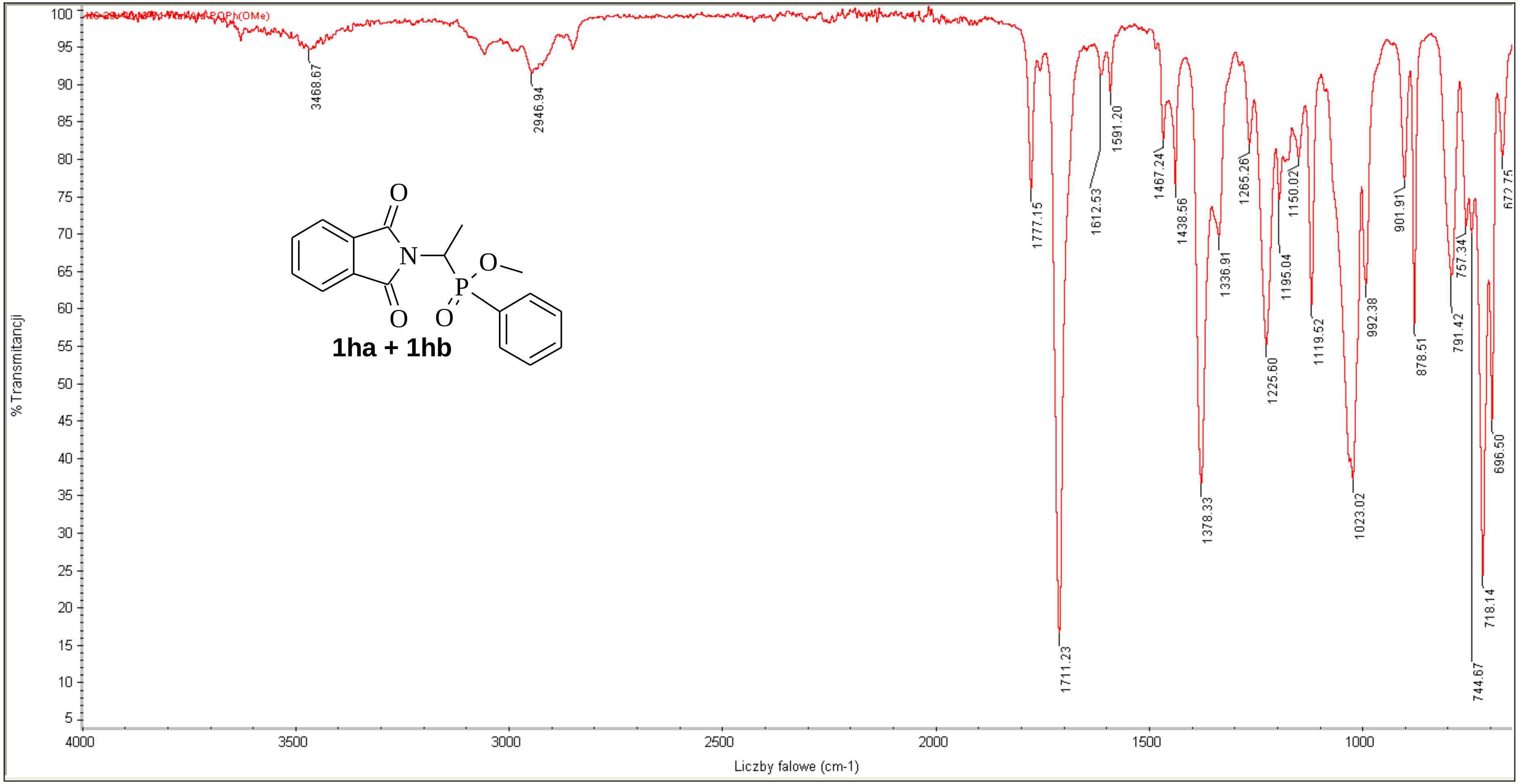

IR spectrum of methyl phenyl[1-(*N*-phthalimido)ethyl]phosphinate - a mixture of two disatereoisomers (**1ha**+**1hb**); ATR, cm^{-1}.

Tolerance = 100.0 mDa / DBE: min = -10.0, max = 200.0
Element prediction: Off
Number of isotope peaks used for i-FIT = 3
Monoisotopic Mass, Even Electron Ions
15 formula(e) evaluated with 7 results within limits (all results (up to 1000) for each mass)
Elements Used:

Mass	RA	Calc. Mass	mDa	PPM	DBE	Formula	i-FIT	i-FIT Norm	Fit Conf %	C	H	N	O	P
330.0898	100.00	330.0895	0.3	0.9	10.5	C17 H17 N O4 P	64.0	2.475	8.41	17	17	1	4	1
		330.1259	-36.1	-109.4	9.5	C18 H21 N O3 P	64.9	3.353	3.50	18	21	1	3	1
		330.0531	36.7	111.2	11.5	C16 H13 N O5 P	63.5	1.992	13.65	16	13	1	5	1
		330.1470	-57.2	-173.3	4.5	C15 H25 N O5 P	62.4	0.929	39.49	15	25	1	5	1
		330.0320	57.8	175.1	16.5	C19 H9 N O3 P	66.1	4.594	1.01	19	9	1	3	1
		330.1834	-93.6	-283.6	3.5	C16 H29 N O4 P	62.7	1.134	32.17	16	29	1	4	1
		329.9956	94.2	285.4	17.5	C18 H5 N O4 P	65.6	4.032	1.77	18	5	1	4	1

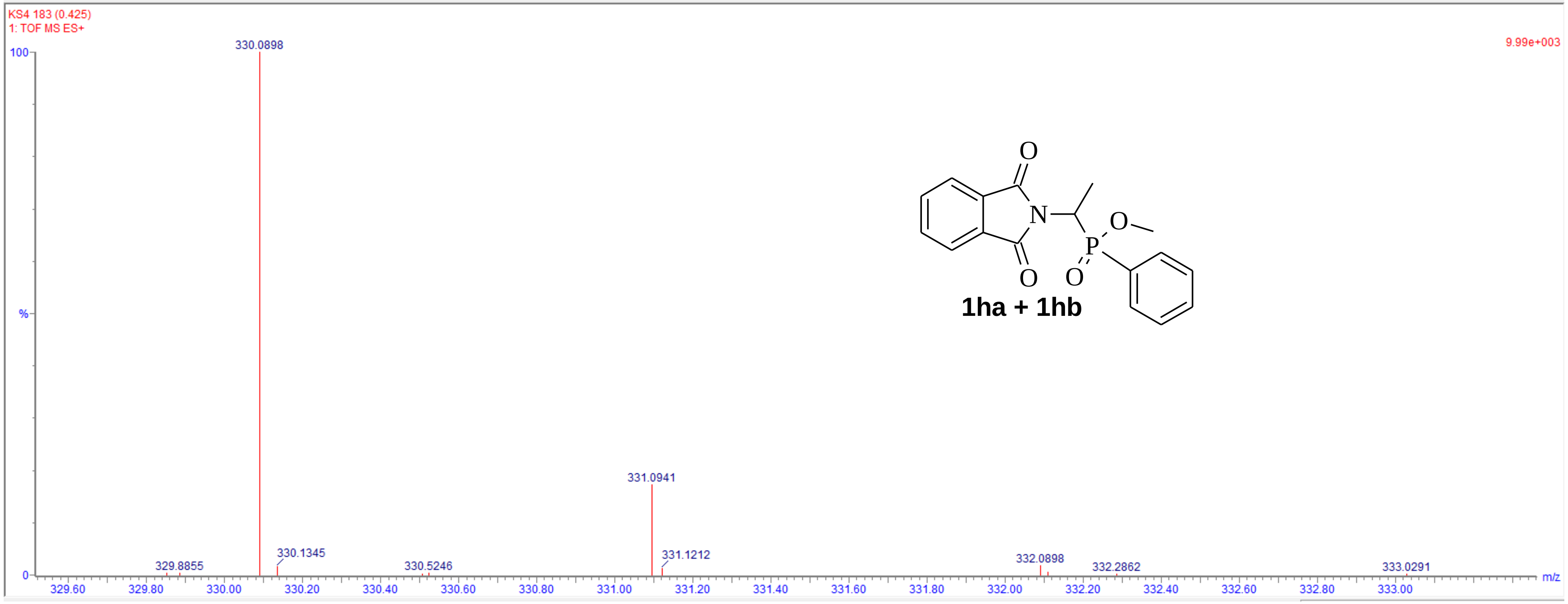

HRMS spectrum of methyl phenyl[1-(*N*-phthalimido)ethyl]phosphinate - a mixture of two disatereoisomers (**1ha**+**1hb**).

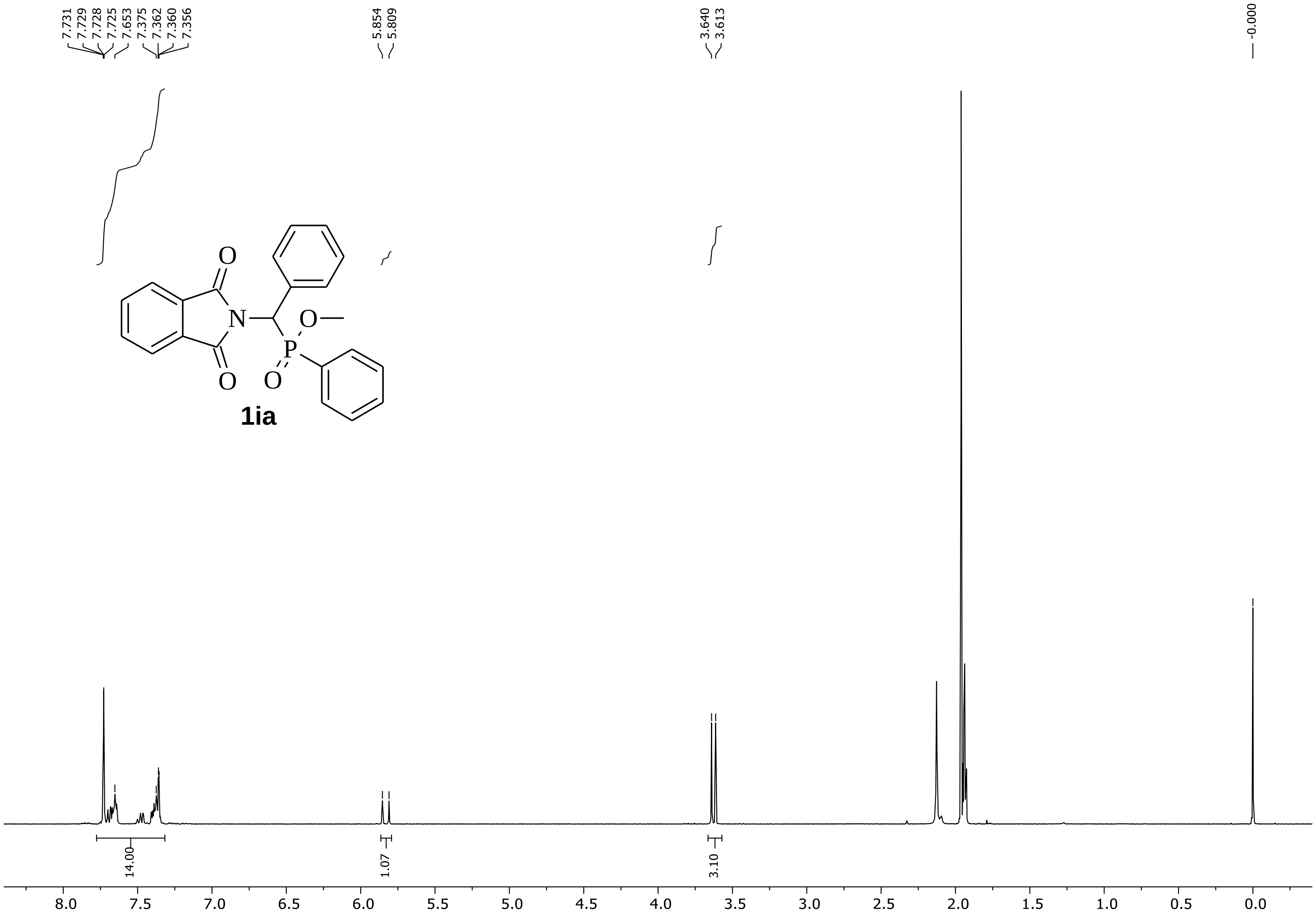

^{1}H-NMR spectrum of methyl phenyl[phenyl(*N*-phthalimido)methyl]phosphinate (**1ia**); 400 MHz/CD_3CN/TMS; δ (ppm) - the first diastereoisomer.

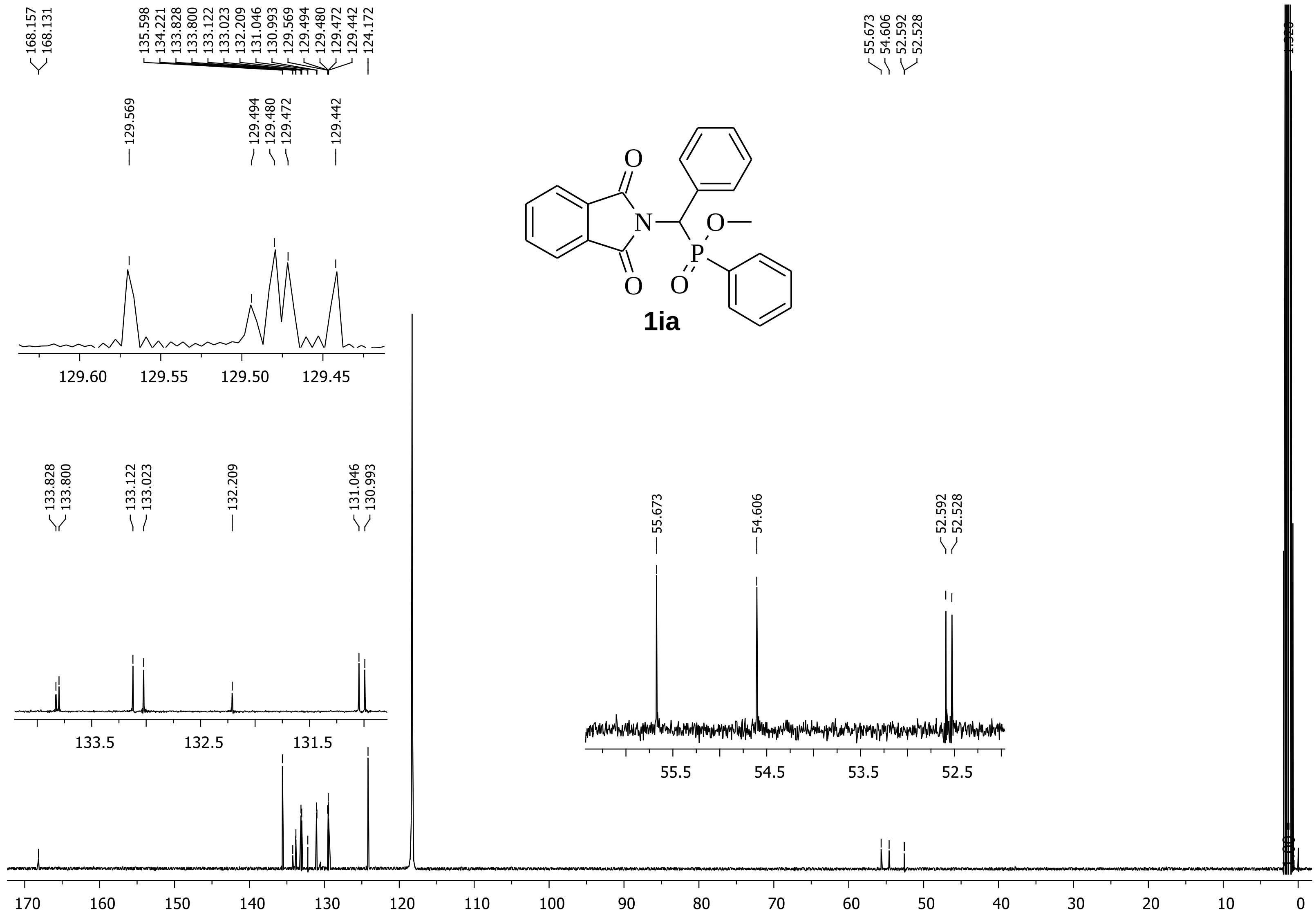

^{13}C-NMR spectrum of methyl phenyl[phenyl(*N*-phthalimido)methyl]phosphinate (**1ia**); 100 MHz/CD_3CN; δ (ppm) - the first diastereoisomer.

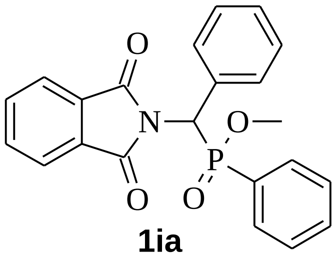

1ia

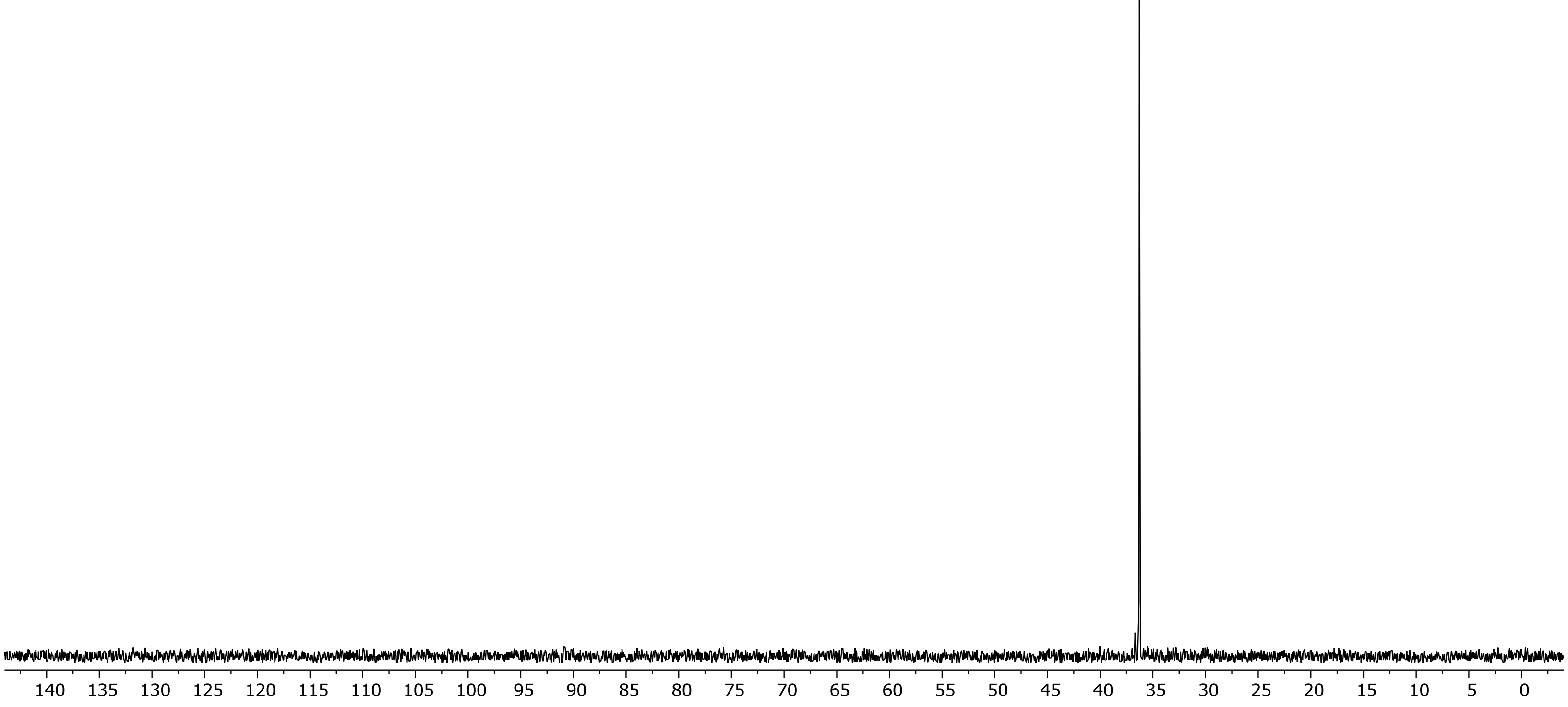

^{31}P-NMR spectrum of methyl phenyl[phenyl(*N*-phthalimido)methyl]phosphinate (**1ia**); 161.9 MHz/CD_3CN; δ (ppm) - the first diastereoisomer.

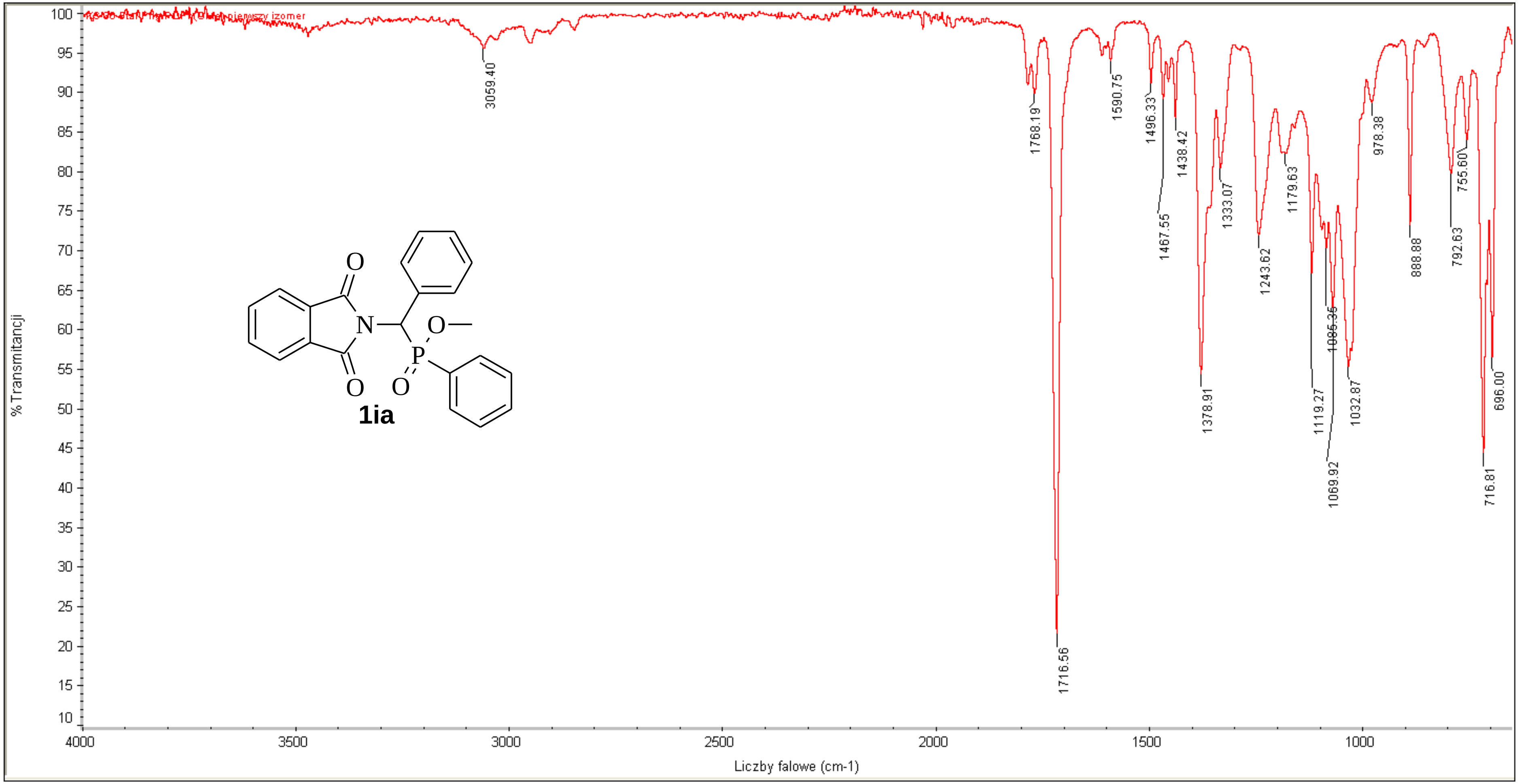

IR spectrum of methyl phenyl[phenyl(*N*-phthalimido)methyl]phosphinate (**1ia**); ATR, cm^{-1}- the first diastereoisomer.

Tolerance = 100.0 mDa / DBE: min = -10.0, max = 200.0
Element prediction: Off
Number of isotope peaks used for i-FIT = 3
Monoisotopic Mass, Even Electron Ions
17 formula(e) evaluated with 7 results within limits (all results (up to 1000) for each mass)
Elements Used:

Mass	RA	Calc. Mass	mDa	PPM	DBE	Formula	i-FIT	i-FIT Norm	Fit Conf %	C	H	N	O	P
392.1054	100.00	392.1052	0.2	0.5	14.5	C22 H19 N O4 P	238.1	0.165	84.75	22	19	1	4	1
		392.1416	-36.2	-92.3	13.5	C23 H23 N O3 P	240.4	2.446	8.66	23	23	1	3	1
		392.0688	36.6	93.3	15.5	C21 H15 N O5 P	242.8	4.873	0.77	21	15	1	5	1
		392.1627	-57.3	-146.1	8.5	C20 H27 N O5 P	244.4	6.442	0.16	20	27	1	5	1
		392.0477	57.7	147.2	20.5	C24 H11 N O3 P	244.4	6.398	0.17	24	11	1	3	1
		392.1991	-93.7	-239.0	7.5	C21 H31 N O4 P	243.2	5.205	0.55	21	31	1	4	1
		392.0113	94.1	240.0	21.5	C23 H7 N O4 P	241.0	3.006	4.95	23	7	1	4	1

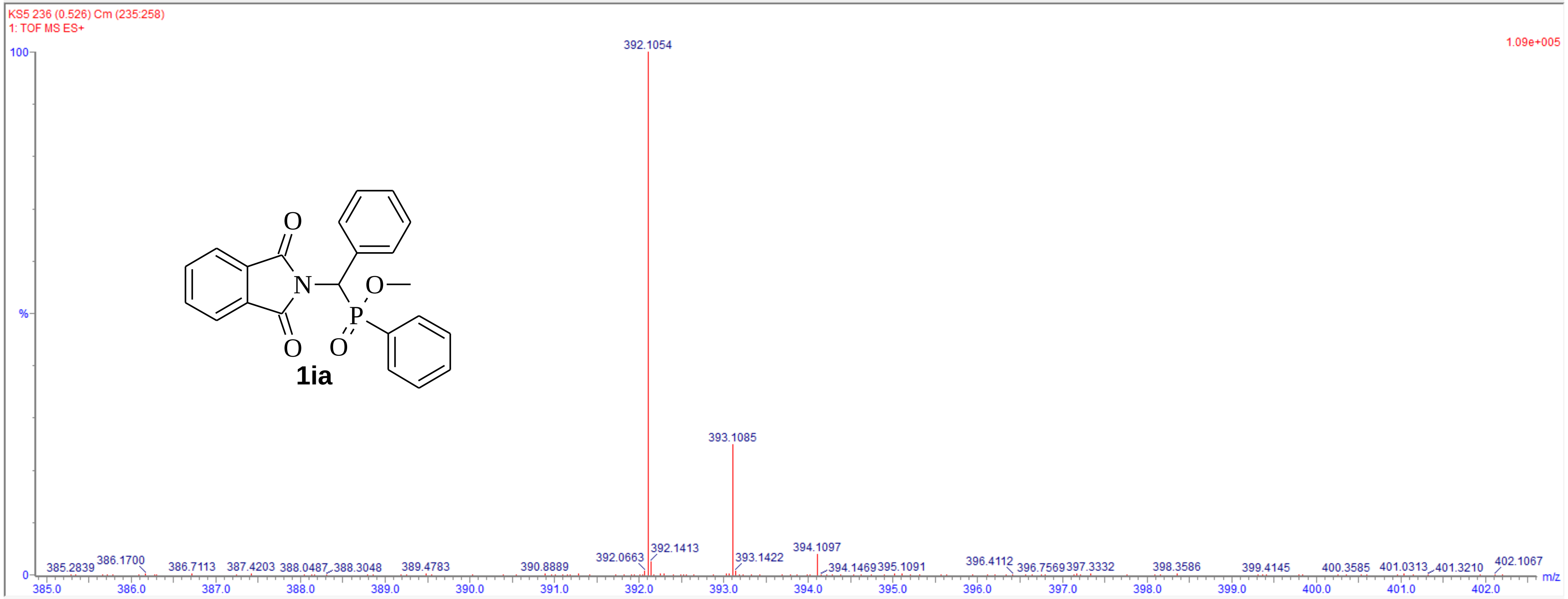

HRMS spectrum of methyl phenyl[phenyl(*N*-phthalimido)methyl]phosphinate (**1ia**) - the first diastereoisomer.

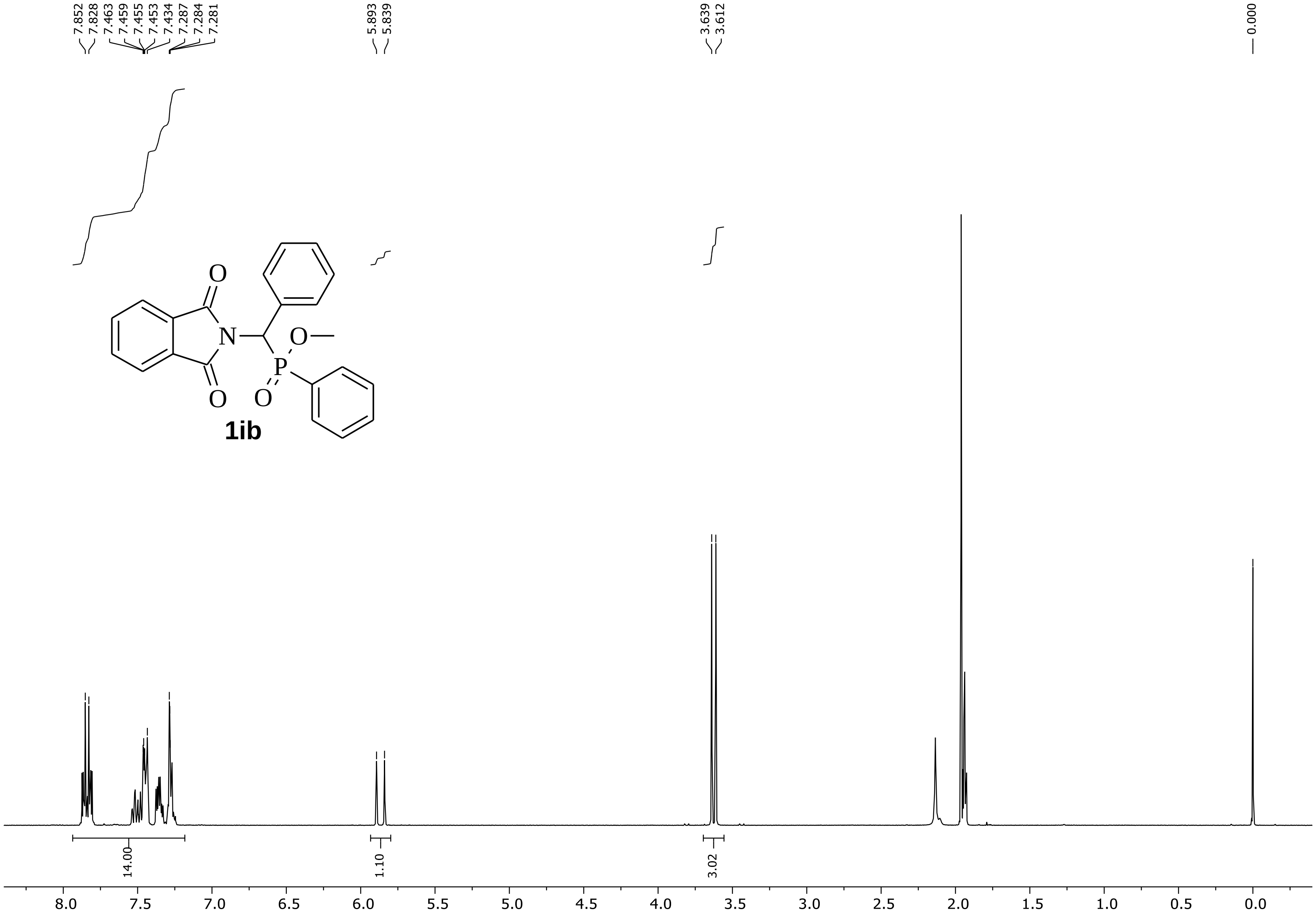

^{1}H-NMR spectrum of methyl phenyl[phenyl(*N*-phthalimido)methyl]phosphinate (**1ib**); 400 MHz/CD_3CN/TMS; δ (ppm) - the second diastereoisomer.

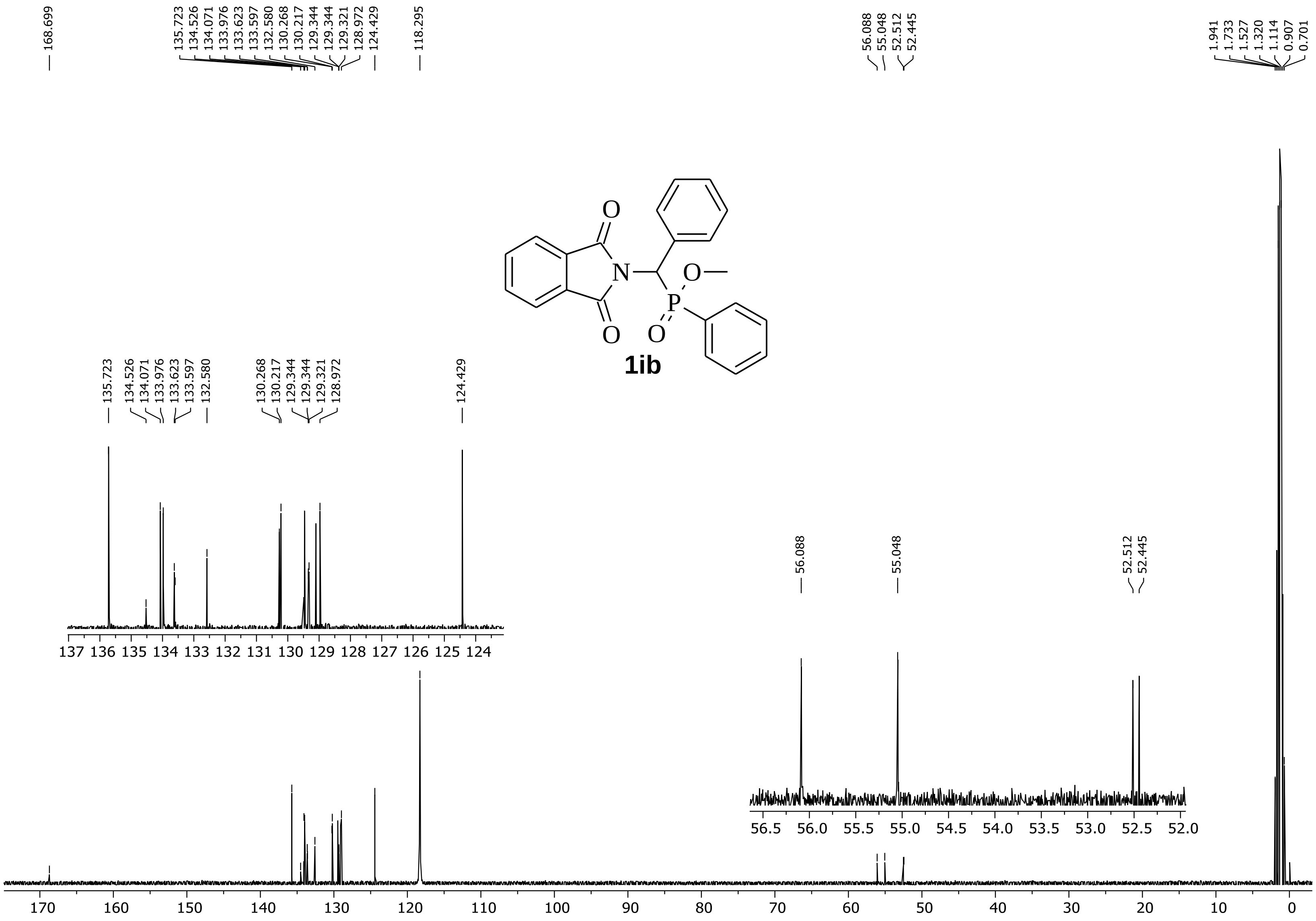

^{13}C-NMR spectrum of methyl phenyl[phenyl(*N*-phthalimido)methyl]phosphinate (**1ib**); 100 MHz/CD_3CN; δ (ppm) - the second diastereoisomer.

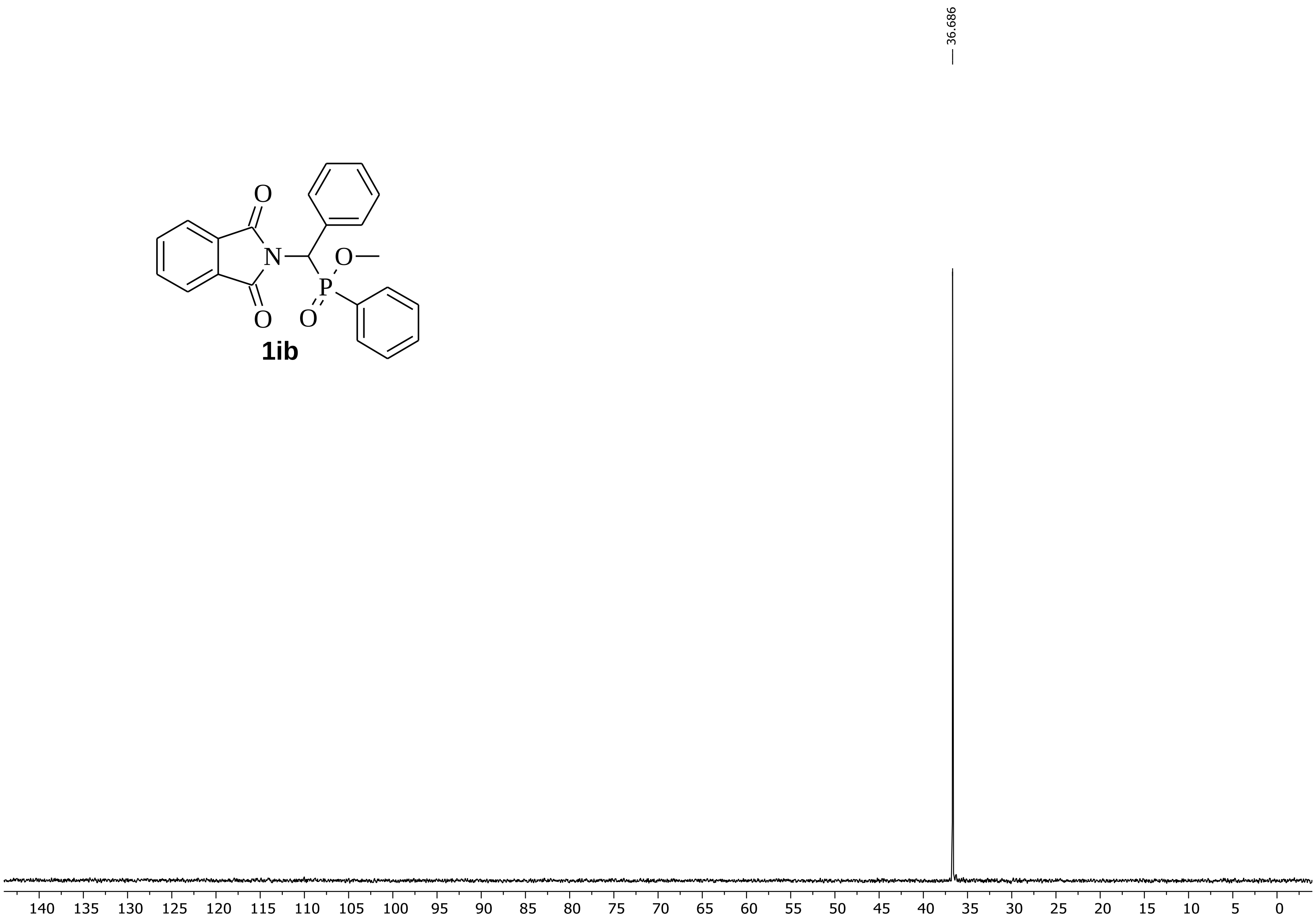

^{31}P-NMR spectrum of methyl phenyl[phenyl(*N*-phthalimido)methyl]phosphinate (**1ib**); 161.9 MHz/CD_3CN; δ (ppm) - the second diastereoisomer.

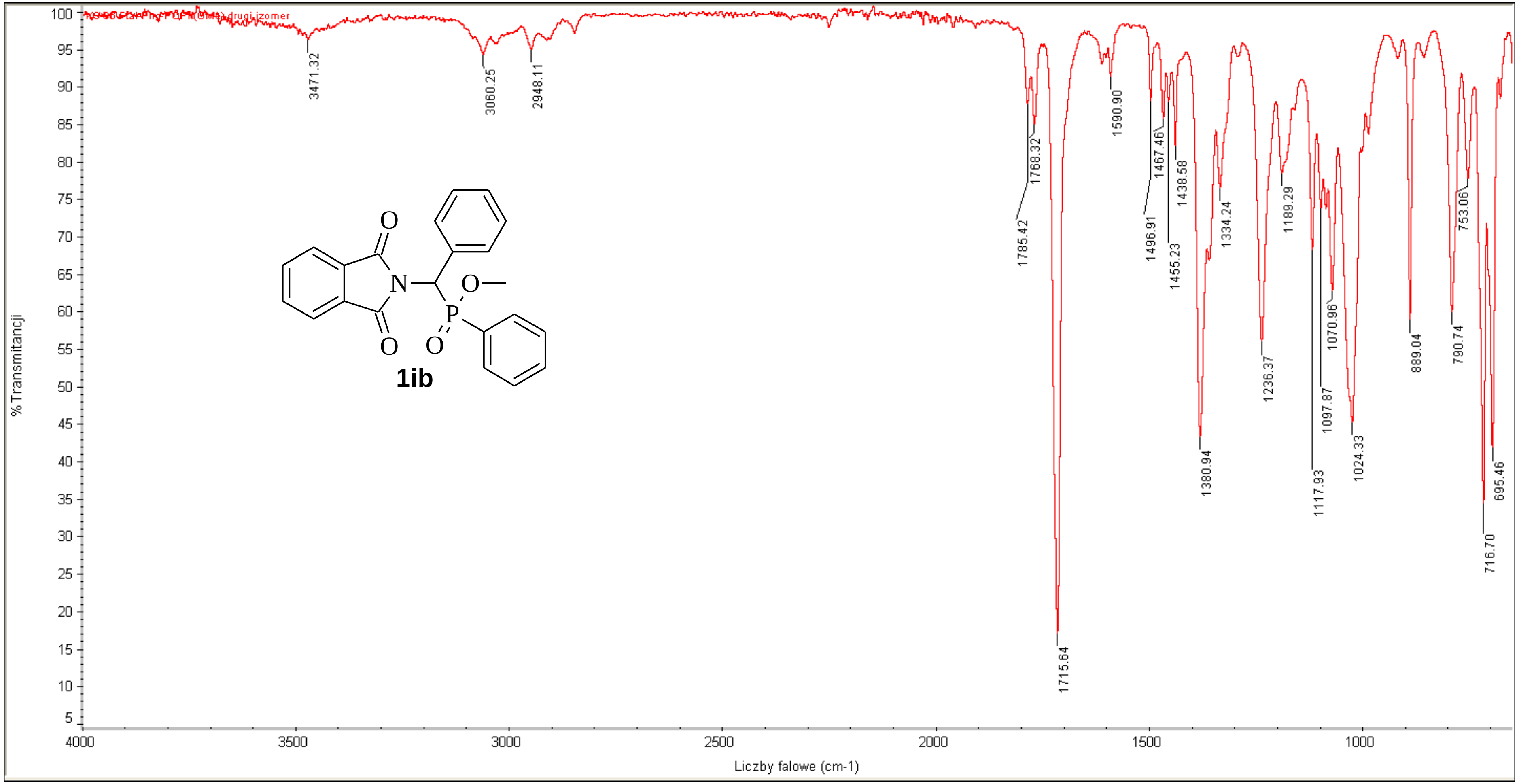

IR spectrum of methyl phenyl[phenyl(*N*-phthalimido)methyl]phosphinate (**1ib**); ATR, cm^{-1}- the second diastereoisomer.

Tolerance = 100.0 mDa / DBE: min = -10.0, max = 200.0
Element prediction: Off
Number of isotope peaks used for i-FIT = 3
Monoisotopic Mass, Even Electron Ions
17 formula(e) evaluated with 7 results within limits (up to 3 closest results for each mass)
Elements Used:

Mass	RA	Calc. Mass	mDa	PPM	DBE	Formula	i-FIT	i-FIT Norm	Fit Conf %	C	H	N	O	P
392.1052	100.00	392.1052	0.0	0.0	14.5	C22 H19 N O4 P	256.7	0.992	37.10	22	19	1	4	1
		392.1416	-36.4	-92.8	13.5	C23 H23 N O3 P	260.3	4.598	1.01	23	23	1	3	1
		392.0688	36.4	92.8	15.5	C21 H15 N O5 P	256.2	0.480	61.90	21	15	1	5	1

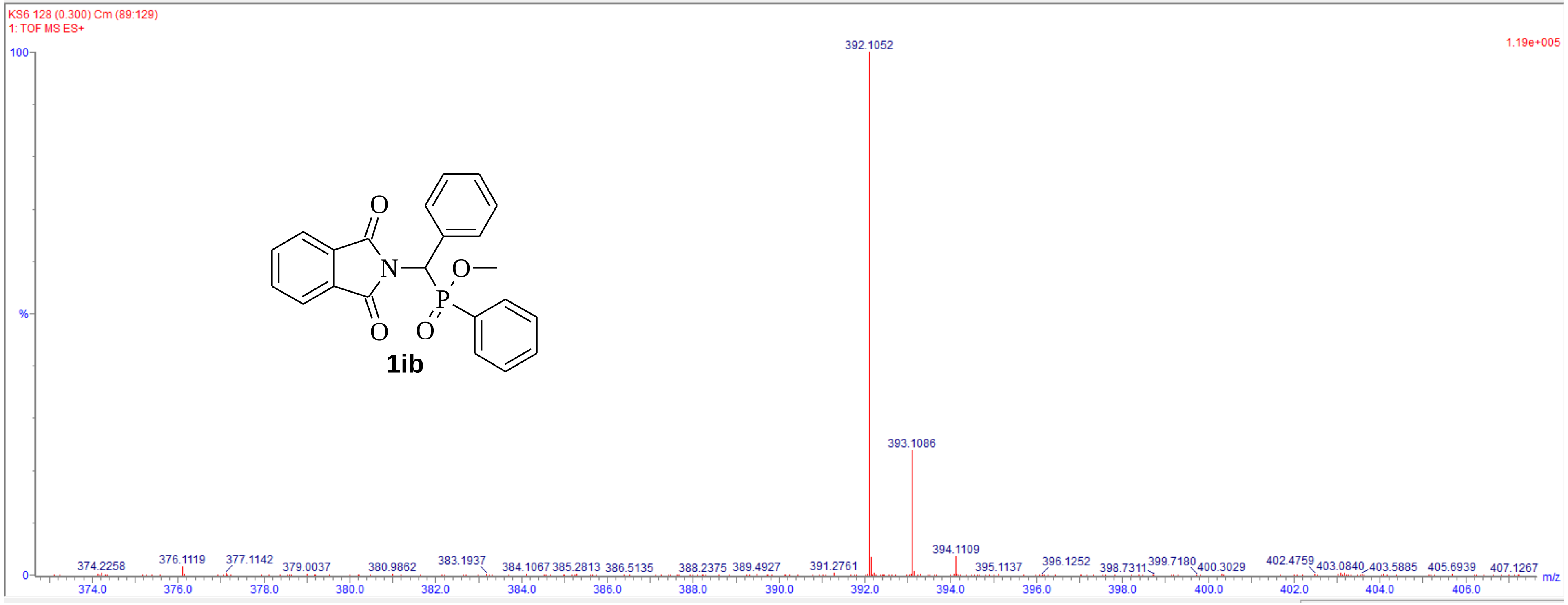

HRMS spectrum of methyl phenyl[phenyl(*N*-phthalimido)methyl]phosphinate (**1ib**) - the second diastereoisomer.

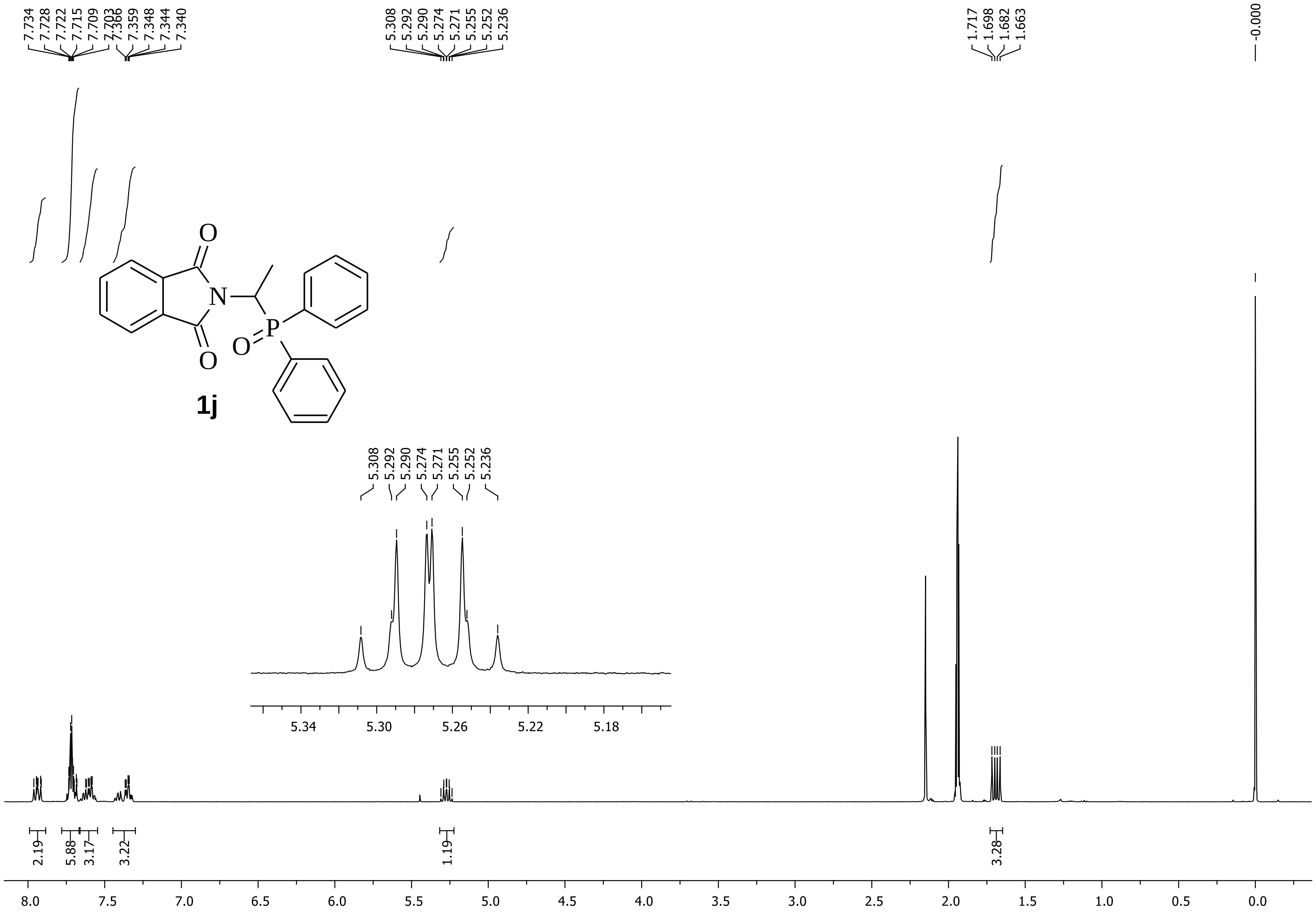

^{1}H-NMR spectrum of diphenyl 1-(*N*-phthalimido)ethylphosphine oxide (**1j**); 400 MHz/CD_3CN/TMS; δ (ppm).

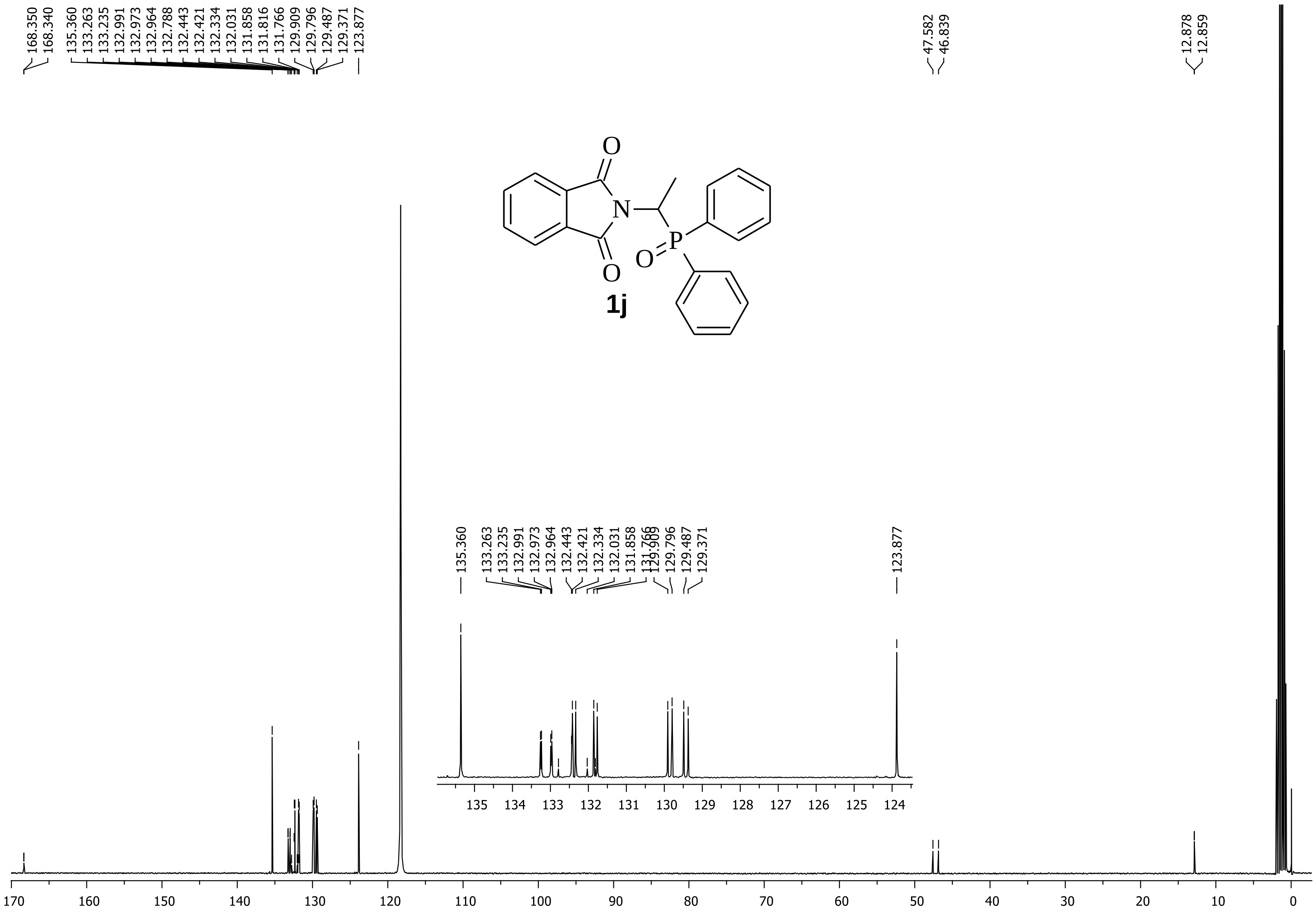

^{13}C-NMR spectrum of diphenyl 1-(*N*-phthalimido)ethylphosphine oxide (**1j**); 100 MHz/CD_3CN; δ (ppm).

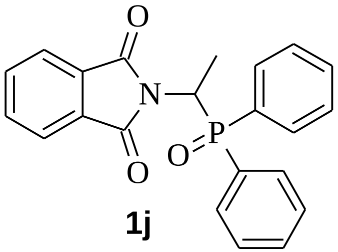

1j

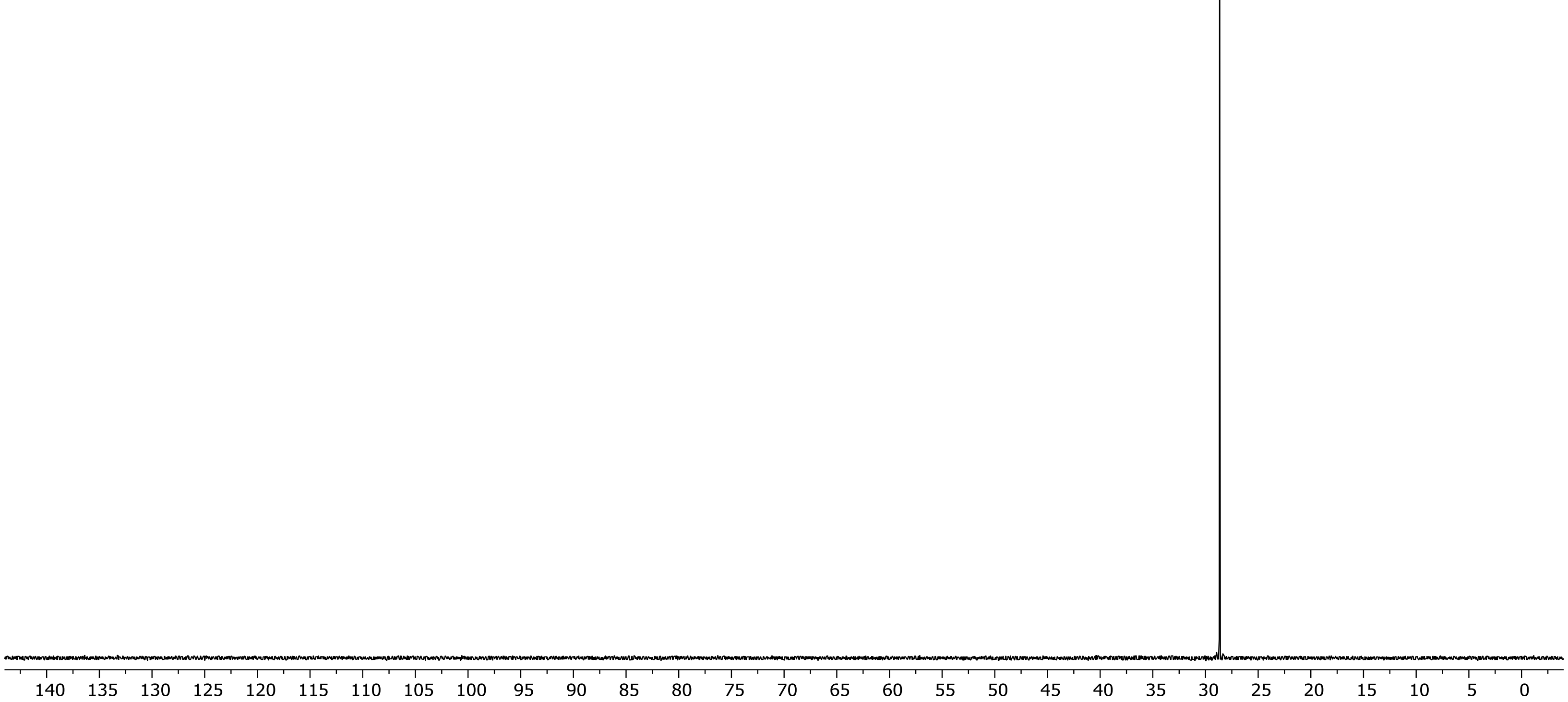

^{31}P-NMR spectrum of diphenyl 1-(*N*-phthalimido)ethylphosphine oxide (**1j**); 161.9 MHz/CD_3CN; δ (ppm).

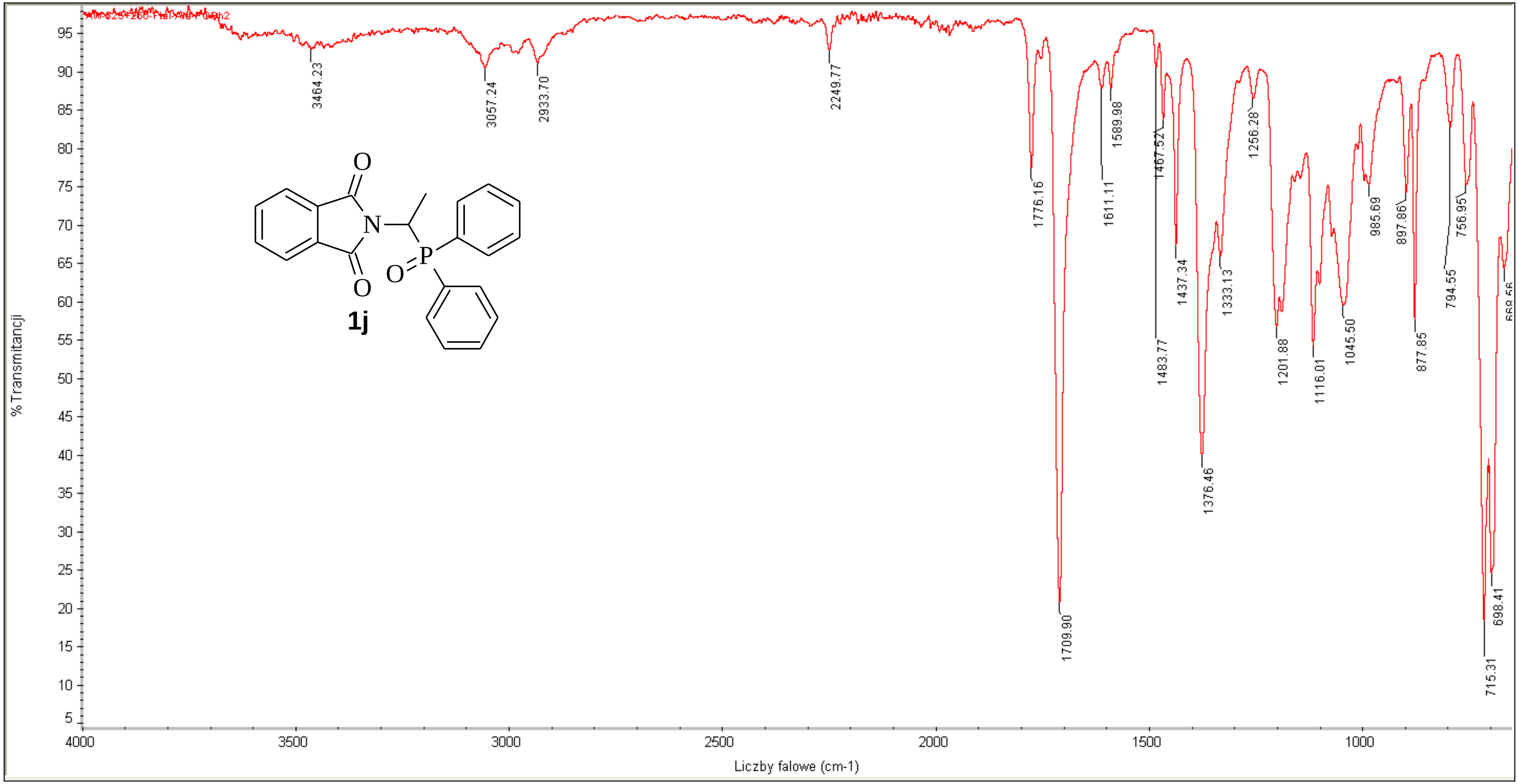

IR spectrum of diphenyl 1-(*N*-phthalimido)ethylphosphine oxide (**1j**); ATR, cm^{-1}.

Tolerance = 100.0 mDa / DBE: min = -10.0, max = 200.0
Element prediction: Off
Number of isotope peaks used for i-FIT = 3
Monoisotopic Mass, Even Electron Ions
6 formula(e) evaluated with 3 results within limits (all results (up to 1000) for each mass)
Elements Used:

Mass	RA	Calc. Mass	mDa	PPM	DBE	Formula	i-FIT	i-FIT Norm	Fit Conf %	C	H	N	O	P
376.1104	100.00	376.1103	0.1	0.3	14.5	C22 H19 N O3 P	527.3	0.010	98.96	22	19	1	3	1
		376.2042	-93.8	-249.4	7.5	C21 H31 N O3 P	535.4	8.074	0.03	21	31	1	3	1
		376.0164	94.0	249.9	21.5	C23 H7 N O3 P	531.9	4.595	1.01	23	7	1	3	1

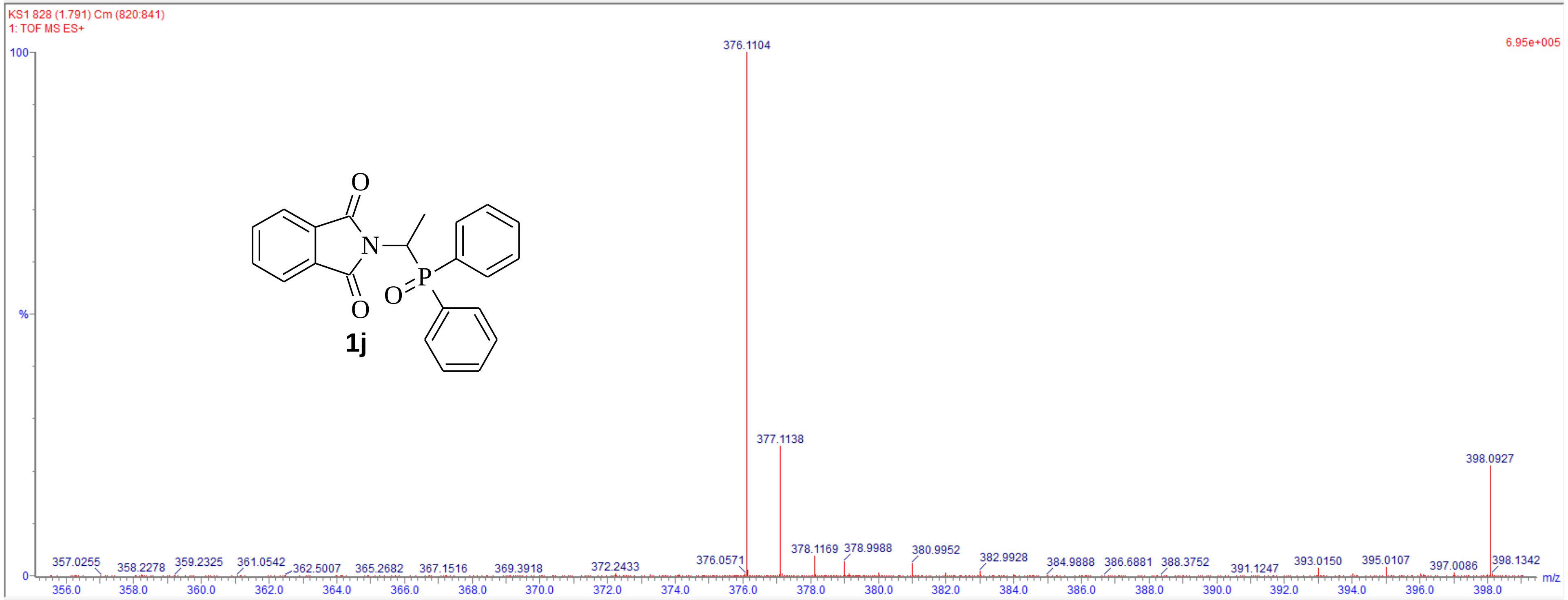

HRMS spectrum of diphenyl 1-(*N*-phthalimido)ethylphosphine oxide (**1j**).

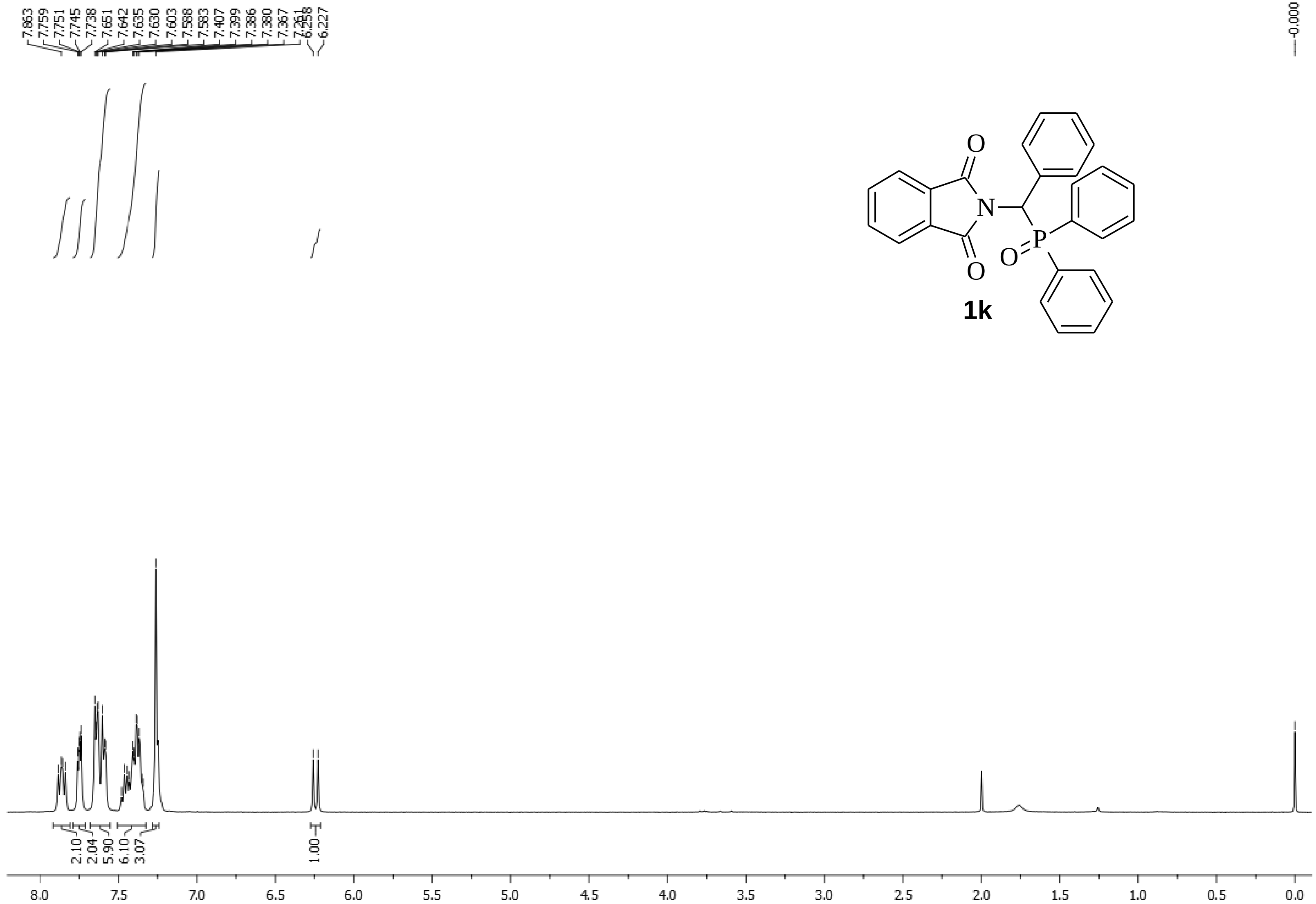

^{1}H-NMR spectrum of diphenyl phenyl(*N*-phthalimido)methylphosphine oxide (**1k**); 400 MHz/$CDCl_3$/TMS; δ (ppm).

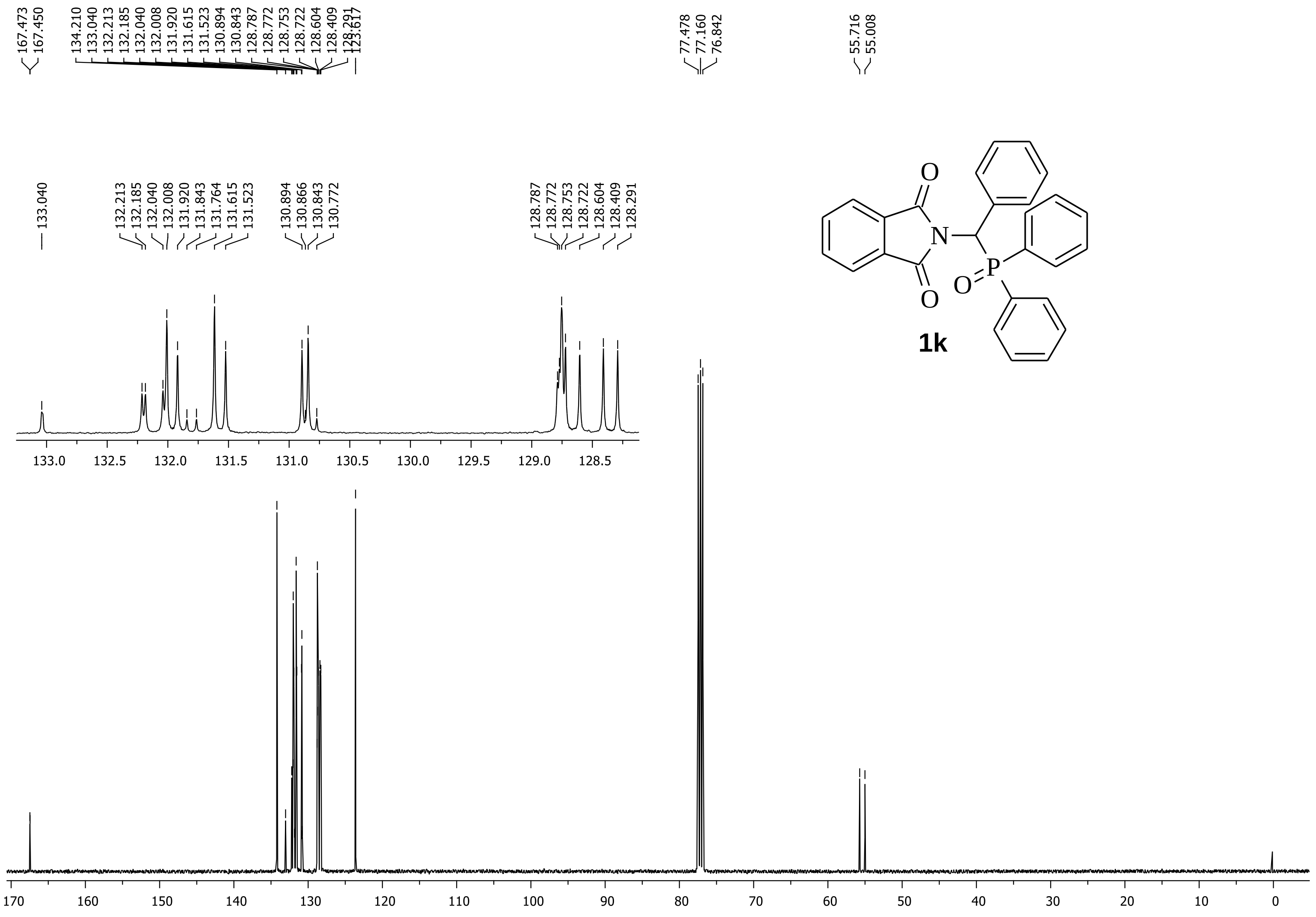

^{13}C-NMR spectrum of diphenyl phenyl(*N*-phthalimido)methylphosphine oxide (**1k**); 100 MHz/CDCl$_3$; δ (ppm).

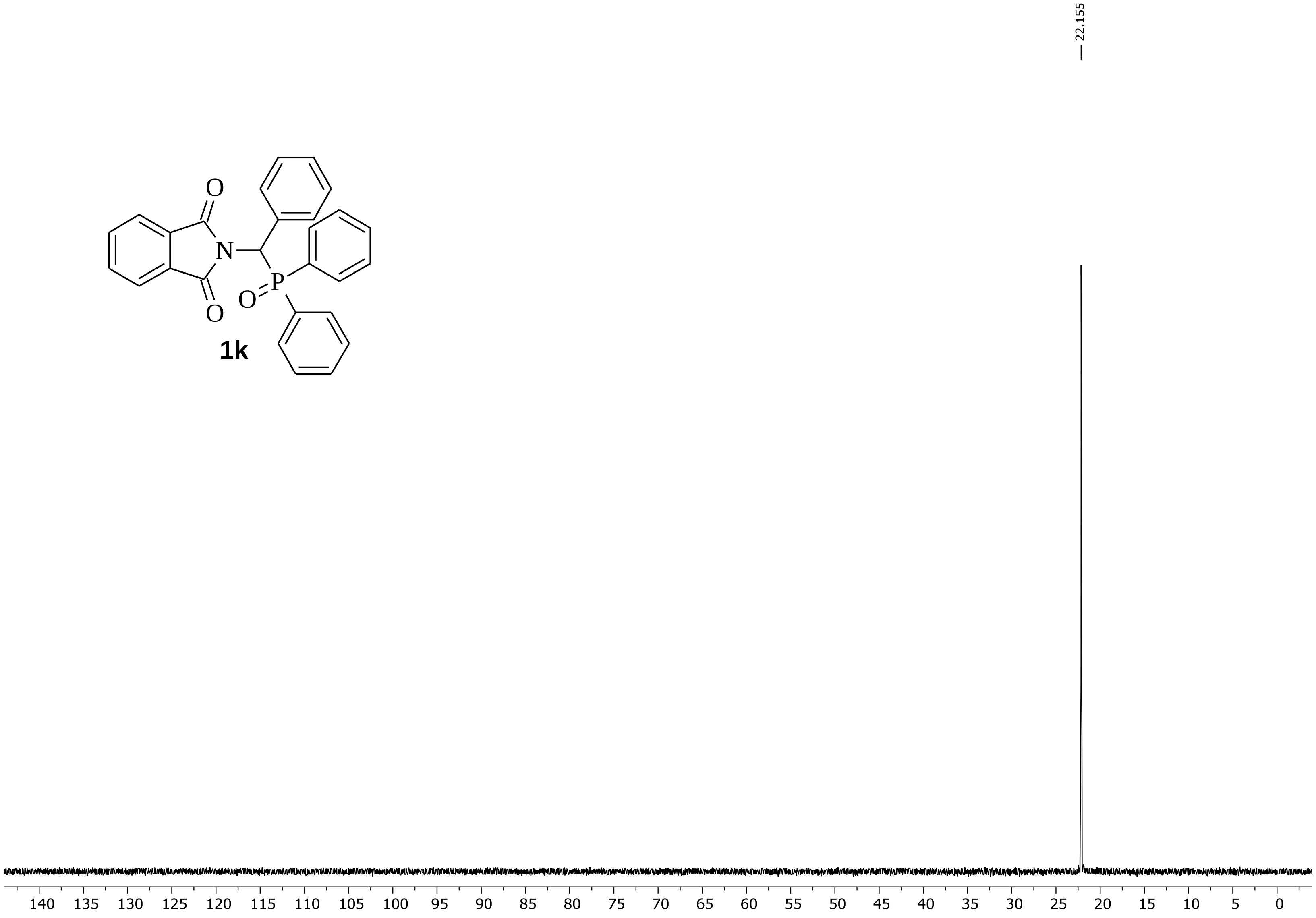

^{31}P-NMR spectrum of diphenyl phenyl(*N*-phthalimido)methylphosphine oxide (**1k**); 161.9 MHz/$CDCl_3$; δ (ppm).

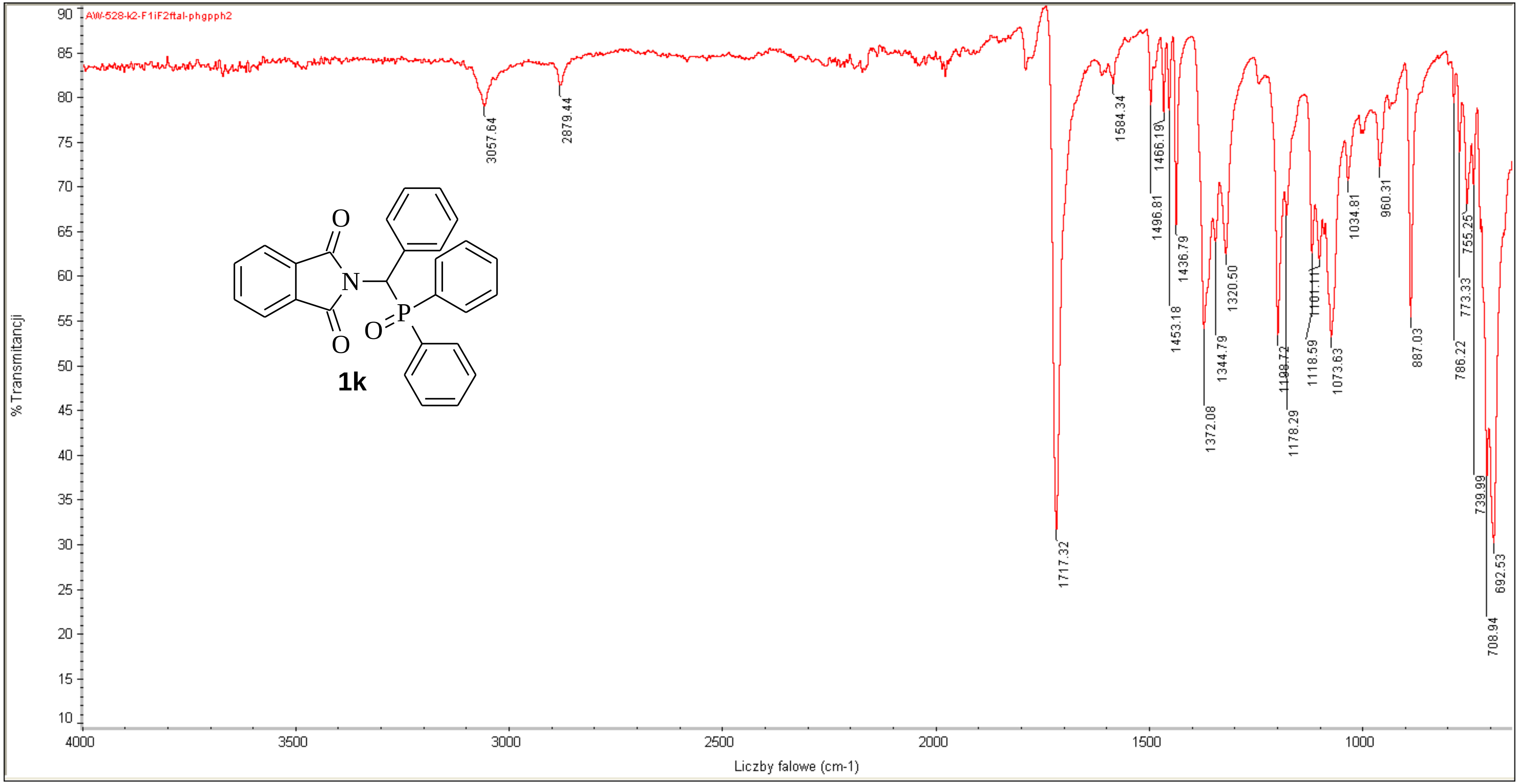

IR spectrum of diphenyl phenyl(*N*-phthalimido)methylphosphine oxide (**1k**); ATR; cm^{-1}.

Tolerance = 100.0 mDa / DBE: min = -10.0, max = 200.0
Element prediction: Off
Number of isotope peaks used for i-FIT = 3
Monoisotopic Mass, Even Electron Ions
6 formula(e) evaluated with 3 results within limits (all results (up to 1000) for each mass)
Elements Used:

Mass	RA	Calc. Mass	mDa	PPM	DBE	Formula	i-FIT	i-FIT Norm	Fit Conf %	C	H	N	O	P
438.1262	100.00	438.1259	0.3	0.7	18.5	C27 H21 N O3 P	639.5	4.235	1.45	27	21	1	3	1
		438.2198	-93.6	-213.6	11.5	C26 H33 N O3 P	635.3	0.015	98.55	26	33	1	3	1
		438.0320	94.2	215.0	25.5	C28 H9 N O3 P	645.9	10.664	0.00	28	9	1	3	1

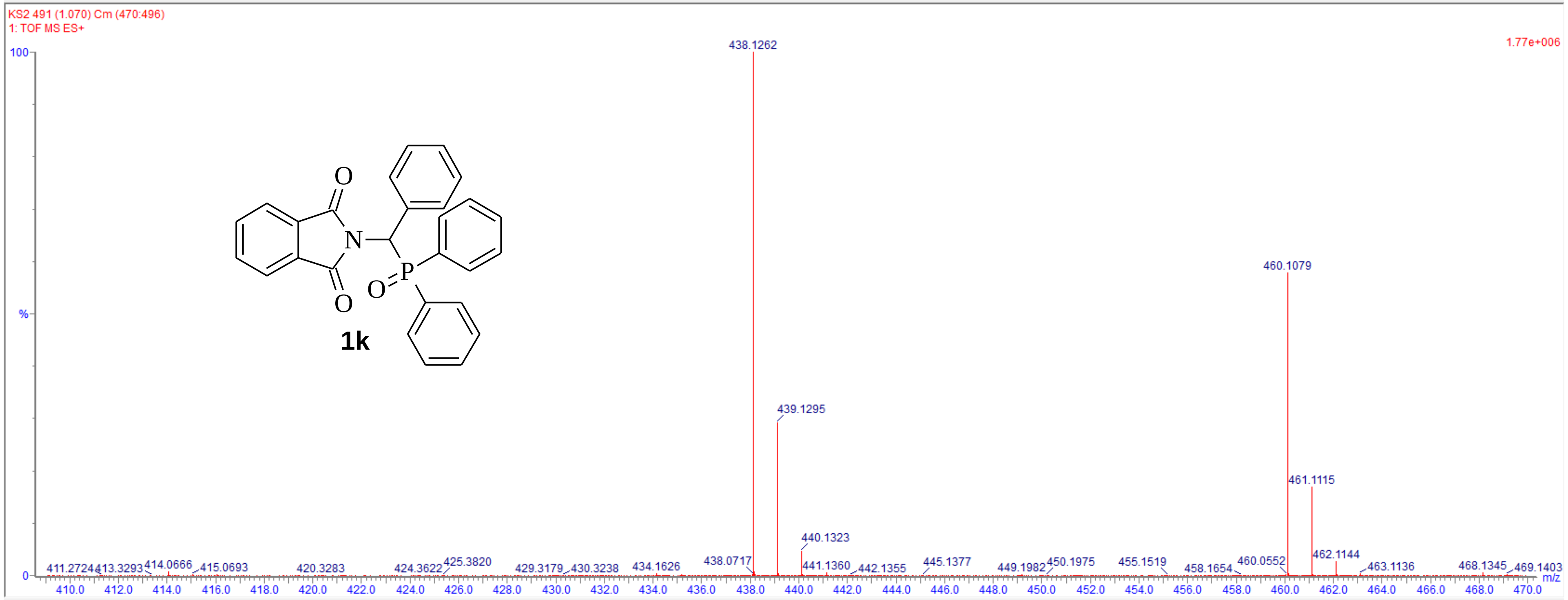

For Help, press F1

HRMS spectrum of diphenyl phenyl(*N*-phthalimido)methylphosphine oxide (**1k**).

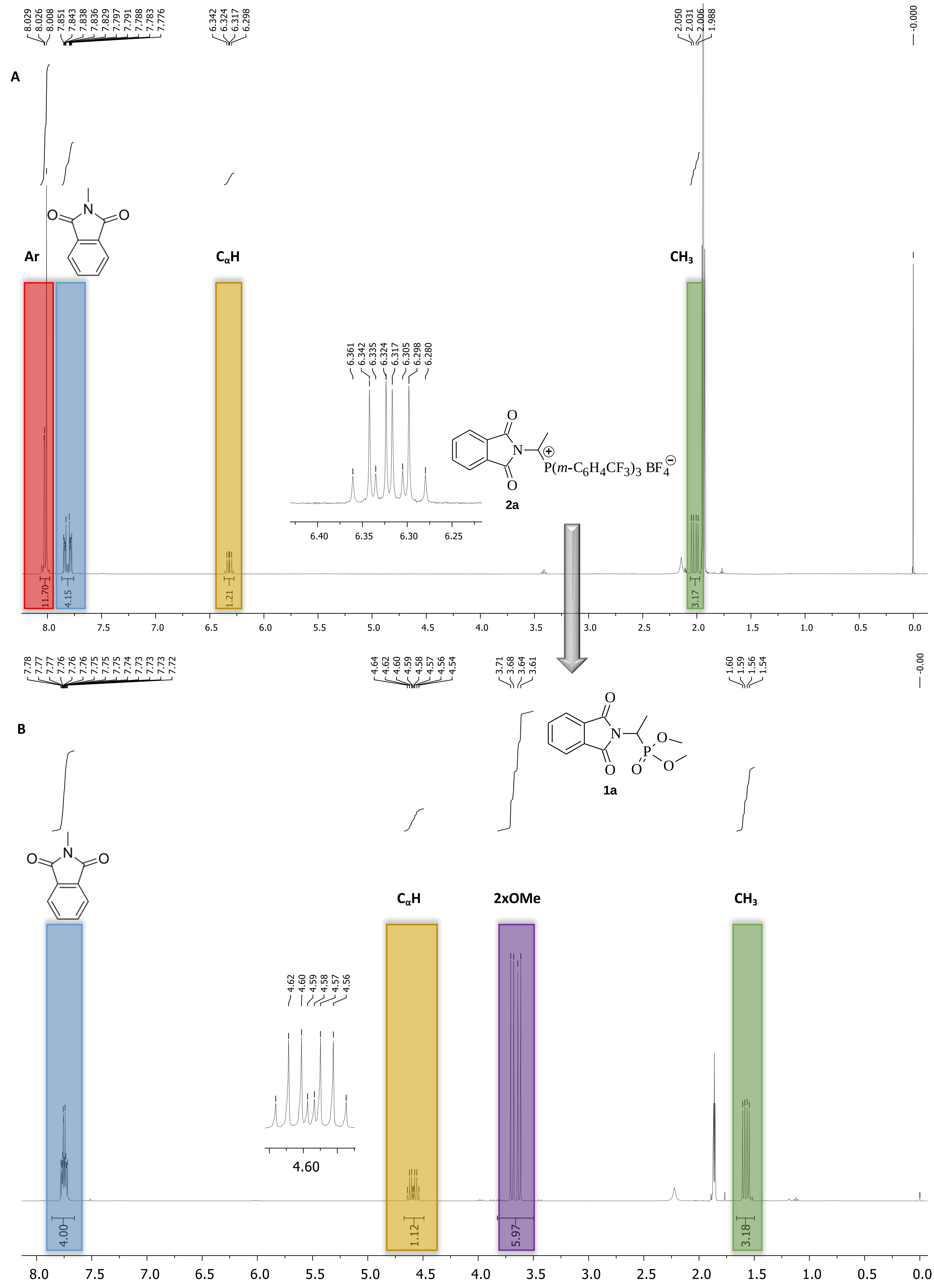

Comparison of ^{1}H-NMR spectra of 1-imidoalkylphosphonium salt **2a** (**A**) and 1-imidoalkylphosphonate **1a** (**B**). The characteristic peaks are marked with colors.
